# A structured lifestyle intervention to reduce cardiometabolic risk factors in individuals with obsessive–compulsive disorder: feasibility trial

**DOI:** 10.1192/bjo.2025.10774

**Published:** 2025-08-01

**Authors:** Anna Holmberg, Matthias Lidin, Dante Lenninger, Sofia Asplund, Catharina Lavebratt, Christian Rück, Lina Martinsson, David Mataix-Cols, Lorena Fernández de la Cruz

**Affiliations:** Centre for Psychiatry Research, Department of Clinical Neuroscience, Karolinska Institutet, Stockholm, Sweden; Stockholm Health Care Services, Region Stockholm, Stockholm, Sweden; Department of Medicine, Karolinska Institutet, Stockholm, Sweden; Department of Cardiology, Heart, Vascular and Neuro Theme, Karolinska University Hospital, Stockholm, Sweden; Department of Molecular Medicine and Surgery, Karolinska Institutet, Stockholm, Sweden; Center for Molecular Medicine, Karolinska University Hospital Solna, Stockholm, Sweden; Department of Clinical Sciences, Lund University, Lund, Sweden

**Keywords:** Obsessive–compulsive disorder, cardiometabolic risk factors, lifestyle intervention, prevention, feasibility trial

## Abstract

**Background:**

Obsessive–compulsive disorder (OCD) is associated with an increased risk of cardiometabolic disorders. We developed a lifestyle intervention, named LIFT, aimed at improving lifestyle habits (physical activity, diet, alcohol and tobacco use, stress, sleep) and reducing cardiometabolic risk factors in OCD.

**Aims:**

This study aimed to establish the feasibility and acceptability of LIFT, evaluate its preliminary efficacy and explore experiences of participation.

**Method:**

Individuals with OCD and at least three cardiometabolic risk factors (e.g. physical inactivity, unhealthy diet, overweight/obesity, dyslipidaemia) were offered LIFT, consisting of one individual session to set individual goals, six educational group sessions and 12 exercise group sessions, delivered over 3 months. We collected baseline, post-intervention and 3-month follow-up measures. Preliminary efficacy variables were analysed with linear mixed models and within-group effect sizes. Qualitative interviews were conducted.

**Results:**

Out of 147 screened individuals, 25 were included (68% women, mean age 37.4, s.d. = 10.9). Credibility and satisfaction were high, attrition rates were low (16%) and the programme was generally safe. Recruitment and adherence to the intervention were challenging. Statistically significant improvements were observed in dietary habits, alcohol consumption, stress, OCD symptom severity and general functioning (within-group effect sizes ranging from 0.27 to 0.56). No changes were observed in physical activity, sleep or any physiological or laboratory measures.

**Conclusions:**

Overall, LIFT was a feasible intervention for individuals with OCD. Effects on lifestyle habits, mental health and functioning are promising. Fully powered randomised controlled trials are needed to evaluate its efficacy and cost-effectiveness.

Obsessive–compulsive disorder (OCD) affects about 1–2% of the population^1^ and is associated with an increased risk of morbidity and mortality caused by endocrine, metabolic and circulatory system disorders, such as cardiovascular diseases, type 2 diabetes, obesity and metabolic syndrome.^2–6^ The reasons behind these associations are still unclear, but the current evidence suggests that they are unlikely to be entirely attributable to familial factors or the use of OCD medication.^2–4^ Instead, the risk is hypothesised to be related to non-shared environmental risk factors such as pernicious lifestyle habits (e.g. low physical activity, unhealthy diet, tobacco and alcohol use).^2–4^ Unhealthy lifestyle habits are linked to the development of cardiometabolic disorders and other somatic issues.^7^ Previous studies showed that individuals with OCD have an increased risk of sleep problems,^8^ smoking^9^ and substance misuse.^10^ Although more sparsely studied, individuals with OCD also self-report low levels of physical activity and unhealthy diets.^11,12^ Lifestyle interventions aimed at reducing cardiometabolic risk have been tested successfully in the general population^13,14^ and in individuals with schizophrenia, bipolar disorder and depression, where the risk of increased morbidity is well established.^15–18^ To our knowledge, no previous studies have targeted the health outcomes of individuals with OCD. However, a few studies have tested the effects of exercise on OCD symptoms, often as an adjunct to cognitive–behavioural therapy (CBT),^19,20^ showing that exercise-based interventions are feasible in individuals with OCD.

To fill this gap, we developed a lifestyle intervention, called LIFT (Swedish acronym for *Livsstilsintervention för tvångssyndrom* (Lifestyle Intervention for OCD)), tailored to individuals with OCD and aimed at improving their lifestyle habits and reducing cardiometabolic risk factors. LIFT considers OCD-specific symptoms (e.g. contamination obsessions, time-consuming rituals) and associated comorbidities (e.g. low mood) that may make it more difficult for people with OCD to implement and maintain changes in their lifestyle. Before designing a fully powered efficacy trial, we tested the feasibility of LIFT in a pilot trial. Our aims were (a) to establish its feasibility and acceptability by measuring ease of recruitment, adherence, credibility, satisfaction, attrition rates and safety; (b) to evaluate its preliminary efficacy 3 months after the intervention and (c) to explore participants’ perspectives and responses to the different components of the intervention to improve the intervention.

## Method

The authors assert that all procedures contributing to this work comply with the ethical standards of the relevant national and institutional committees on human experimentation and with the Helsinki Declaration of 1975, as revised in 2013. All procedures were approved by the Swedish Ethical Review Authority (registration number 2022-00375-01). Written informed consent was provided by all participants. The study was registered in Open Science Framework (https://osf.io/wmxbp). This report follows the Consolidated Standards of Reporting Trials (CONSORT) extension for pilot and feasibility trial guidelines.^21^

### Trial design

This feasibility study used an open trial design with no control group. This design was selected to refine procedures and guide critical decisions before conducting a fully powered randomised controlled trial (RCT). The study protocol can be found in the Supplementary Material, available at https://doi.org/10.1192/bjo.2025.10774.

### Participants and eligibility criteria

Participants were recruited from adult mental health clinics in Stockholm, the local OCD patient organisation (*OCD-föreningen*) and paid social media advertisements.

Inclusion criteria were as follows: (a) being 18 years or older; (b) an OCD diagnosis, based on the DSM-5 criteria and (c) having at least three cardiometabolic risk factors out of a predefined list (see Table 1). Exclusion criteria were as follows: (a) inability to understand Swedish; (b) inability to travel to Stockholm for the intervention; (c) inability to consistently attend the intervention; (d) impairing OCD symptoms that could interfere with participation; (e) intellectual disability or a diagnosis of a psychiatric disorder that could interfere with the intervention (e.g. acute psychosis, severe depression); (f) a diagnosis of an eating or alcohol or drug use disorder; (g) suicide risk; (h) being pregnant or breastfeeding; (i) myocardial infarction or stroke within the previous 6 months; (j) cardiometabolic risk measures that contraindicated participation (e.g. systolic blood pressure ≥180 mmHg or diastolic ≥110 mmHg) or (k) initiation or adjustment of any cardiometabolic medication within the previous 3 months.

**Table 1:** List of cardiometabolic risk factors and corresponding operational definitions used as inclusion criteria

#	Cardiometabolic risk factors	Operational definition
1.	Risk alcohol consumption	>9 units/week or >4 units/occasion for women, >14 units/week or >5 units/occasion for men^22^
2.	Physical inactivity	<150 min activity/week moderate intensity (i.e. heart beats faster, breathing is faster but able to talk) or <75 min high intensity (i.e. pulse is high, shortened breath, difficulties talking)^23^
3.	Unhealthy diet	A score of ≤4 points on the dietary questionnaire developed by the Swedish National Board of Health and Welfare^22^
4.	Tobacco use (smoking/snuff)	>1 cigarette/day
5.	Current or previous cardiovascular disorder	Except for myocardial infarction or stroke within the past 6 months. Ascertained by self-report in telephone screening and at the initial cardiometabolic assessment
6.	Abdominal obesity	Waist circumference >88 cm in women, >102 cm in men, according to criteria from the World Health Organization (2011) or SAD: >20 cm in women and >22 cm in men^24^
7.	Overweight or obese	Body mass index >25 according to criteria from the World Health Organization^24^
8.	Hypertension	Systolic blood pressure over 140 mmHg and diastolic blood pressure over 90 mmHg or currently on medication for hypertension^24^
9.	Dyslipidemia	Elevated total or LDL cholesterol levels or as a high ratio between LDL and HDL. Total >5.0 mmol/L, LDL >3.0 mmol/L, at-risk populations LDL >1.4 mmol/L^24^
10.	Impaired glucose tolerance (prediabetes)	Reference levels for impaired glucose tolerance is between ≥7.8 and <11.1 mmol/L according to World Health Organization criteria^25^
11.	Type 2 diabetes mellitus	Previous clinical diagnosis or fasting blood glucose levels over 7.0 mmol/L or an OGTT with a 2-h level of 11.1 mmol/L or over^25^

SAD, sagittal abdominal diameter; LDL, low-density lipoprotein; HDL, high-density lipoprotein; OGTT, oral glucose tolerance test.

### Study procedures

Individuals expressing interest were first contacted via telephone for an initial eligibility screening. If suitable, they were invited to a psychiatric assessment of approximately 1–1.5 h duration, conducted by the study coordinator (A.H.), a clinical psychologist expert in the assessment and treatment of OCD. A diagnosis of OCD was confirmed using the Structured Clinical Interview for DSM-IV,^26^ adapted by the research group to align with the DSM-5 criteria for OCD and related disorders. Psychiatric comorbidity and suicide risk were assessed with the Mini-International Neuropsychiatric Interview.^27^ OCD severity was measured with the Yale–Brown Obsessive–Compulsive Scale (Y-BOCS).^28^

After the psychiatric assessment, potential participants still considered eligible were invited to a cardiometabolic assessment at the Department of Cardiology at Karolinska Hospital, Solna, Sweden, to confirm that cardiometabolic risk was present, and, if so, collect baseline measurements. A cardiovascular nurse collected the data through a semi-structured interview, anthropometric measurements and blood tests. Participants who met all eligibility criteria after this visit were included in the study. At this point, they also completed the remaining baseline questionnaires.

Anthropometric measurements, blood tests and self-reported questionnaires were repeated at post-intervention and 3 months after the intervention. At these time points, the study coordinator (A.H.) also contacted the participants on the telephone to re-assess OCD symptom severity, using the Y-BOCS.

### Outcome measures

#### Aim 1: feasibility and acceptability

The primary outcomes were feasibility and acceptability, evaluated measuring different constructs. Ease of recruitment was assessed by exploring the recruitment rate, number of exclusions and reasons, and number of individuals who declined participation and their reasons. Adherence was measured by counting the number of sessions attended. To assess intervention credibility, a short self-reported questionnaire developed by the research team was used at baseline, consisting of three items scored on a five-point Likert scale (0–4). Intervention satisfaction was measured at post-intervention with the self-reported Client Satisfaction Questionnaire-8 items (CSQ-8).^29^ Each item is scored on a four-point Likert scale (8–32). Attrition rates were measured by counting number of drop-outs and, when possible, exploring reasons for disengaging from the intervention. Safety was measured with a self-reported questionnaire developed by the research team aiming at monitoring adverse events at mid- and post-intervention. Additionally, participants could write their own symptoms. Adverse events were also monitored by the group leaders at the educational and exercise sessions and in the brief follow-up telephone call between sessions.

#### Aim 2: preliminary efficacy

To assess preliminary efficacy, several measures were collected at baseline, post-intervention and 3-month follow-up (primary end-point). An extended description of the self-reported preliminary efficacy measures, including psychometric properties, can be found in the Supplementary Materials.

##### Lifestyle habits

Level of physical activity and sedentary behaviour were measured with the seven-item self-reported International Physical Activity Questionnaire (IPAQ), short form.^30^ Participants also carried a hip-worn accelerometer (ActiGraph GT3X-BT) for seven consecutive days. This was activated at the cardiometabolic assessment visit at the three different time points. Dietary habits were measured by a five-item questionnaire covering consumption of vegetables, fruit, fish, sweets/snacks and breakfast habits.^22^ Alcohol consumption was measured with the three-item self-reported Alcohol Use Disorder Identification Test for Consumption (AUDIT-C).^31^ Tobacco use was evaluated by asking the participant about current or previous cigarette/snuff use, and, if applicable, the amount of cigarettes/snuff used. For the analysis, smokers and snuff users were merged together. Stress was measured with the ten-item Perceived Stress Scale (PSS-10).^32^ Sleep was measured with the seven-item self-reported Insomnia Severity Scale (ISI).^33^

##### Anthropometric measurements and blood samples

Weight was measured to the nearest 0.1 kg. Body mass index (BMI) was calculated by dividing weight by height squared. Waist circumference was measured in a standing position, midway between the lower rib margin and the iliac crest. Sagittal abdominal diameter was measured in a supine position at the nearest 0.1 cm. Total body fat and fat around the waist in relation to body mass measurements were conducted by using a bio-impedance scale. Systolic and diastolic blood pressure were measured in a seated position, after 5 min of rest, with a standard sphygmomanometer, according to gold standards.^34^ Resting heart rate was measured in a seated position following a 5-min rest.

Total cholesterol, low-density lipoprotein (LDL) cholesterol, high-density lipoprotein (HDL) cholesterol, triglycerides, glucose, glycated haemoglobin (HbA1c) and C-reactive protein were analysed from blood collected after a minimum of 10 h fast. Further, at baseline and 3-month follow-up, an oral glucose tolerance test was performed where glucose levels were measured twice in venous blood, 2 h apart, with HemoCue®, except for participants already diagnosed with type 2 diabetes. Blood samples to analyse inflammatory markers, telomere length and telomerase activity were also collected and stored at the Region Stockholm Biobank. Data on the biobank samples will be reported separately.

The Framingham risk score estimating the 10-year cardiovascular risk of the participants was calculated based on age, gender, smoking status, systolic blood pressure and medical treatment for hypertension, total cholesterol, HDL cholesterol and occurrence of type 2 diabetes.^35^

##### Mental health and quality of life

OCD symptom severity was measured with the clinician-rated Y-BOCS,^28^ consisting of ten items. Additionally, the participants answered the 12-item Obsessive–Compulsive Inventory (OCI-12),^36^ measuring self-reported OCD symptom severity; the Patient Health Questionnaire (PHQ-9), a nine-item scale measuring depressive symptoms during the past 2 weeks;^37^ the Work and Social Adjustment Scale (WSAS),^38^ a five-item scale measuring impairment in functioning; and the EQ-5D-3L,^39^ measuring quality of life and health status in five dimensions, resulting in a health index score, and a health status visual analogue scale.

#### Aim 3: participants’ experiences

Participants’ experiences of participation were explored in a semi-structured qualitative interview on a telephone/video call or face to face. The interview consisted of a list of predefined questions compiled by the research group, divided in two parts. The first part explored the experiences of participation in the trial to be able to further improve the lifestyle intervention (see questions in the Supplementary Materials). The second part focused on how OCD symptoms affect lifestyle habits, the ability to make lifestyle changes and experiences of seeking and receiving somatic healthcare. Results of this second part are presented in a separate report.^40^

All participants that completed the intervention were offered to participate in the qualitative interviews. Interviews were conducted by A.H., D.L. and S.A. For the first set of questions, the interviews were conducted at post-intervention, for the first two groups, or at the 3-month follow-up, for the last two groups.

### Lifestyle intervention

LIFT was developed by the research team. It included evidence based components from a previously evaluated intervention for individuals from the general population with an increased cardiometabolic risk^14^ and group exercise sessions based on the *Braining* project developed at Psychiatry Southwest, Region Stockholm (see below).^41^ All intervention components were adapted to suit the specific needs of individuals with OCD. The team has extensive OCD research and clinical expertise, which guided the OCD-specific adaptations. To ensure that the content of the intervention was appropriate and acceptable for individuals with OCD, members of the Swedish OCD patient organisation (*OCD-förbundet*) and their local group in Stockholm (*OCD-föreningen*) took part in focus groups to provide feedback on the intervention and the study protocol. Their suggestions were incorporated.

**Fig. 1:**
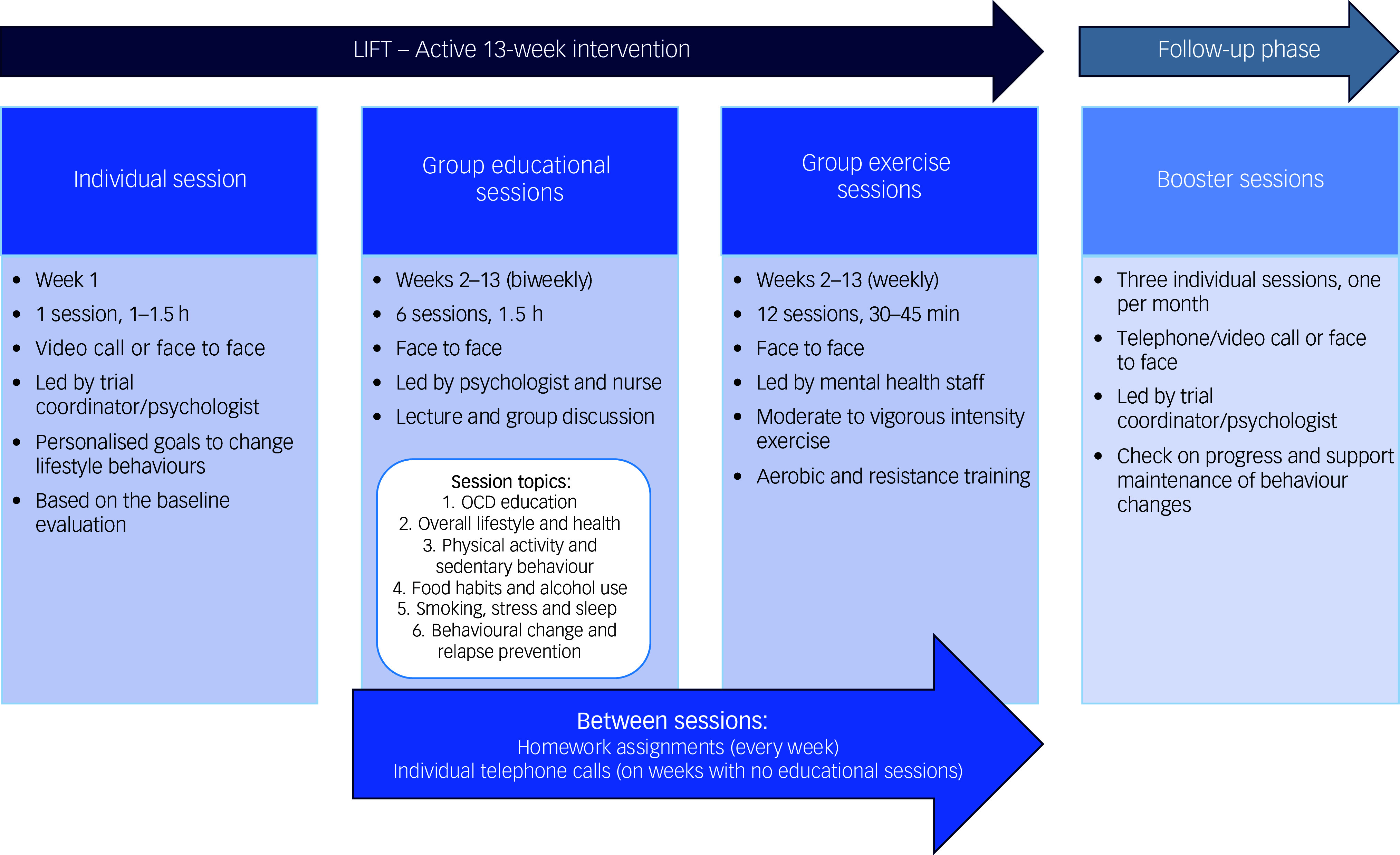
Overview of the LIFT (Swedish acronym for *Livsstilsintervention för tvångssyndrom* (Lifestyle Intervention for OCD)) intervention. OCD, obsessive–compulsive disorder.

The final version of LIFT (Fig. 1) consisted of three components: one individual session to set individual goals, six educational group sessions and 12 exercise group sessions, delivered throughout 3 months (13 weeks). After the baseline assessment, results from the anthropometric assessment and blood samples of each included participant were discussed in a group including the trial coordinator (A.H.), a nurse specialised in cardiovascular disorders and lifestyle habits (M.L.), and the research nurse that performed the assessment to prepare for the individual session. The individual session was done on week 1, led by the trial coordinator (A.H.), and aimed at creating a personalised plan to change lifestyle habits, based on the initial evaluation.

The six 1.5-h manualised educational group sessions were delivered every other week, from week 2, at the specialist OCD clinic (*OCD-programmet*) at Psychiatry Southwest. Sessions were led by at least two of the following research study members: the trial coordinator (A.H.), a nurse specialised in cardiovascular disorders and lifestyle habits (M.L.), and a clinical psychologist in training (D.L). Each session consisted of a lecture on a specific topic and group discussions. Between sessions, participants were assigned homework and received an individual follow-up telephone call. An outline of these sessions is presented in Supplementary Table 1.

The exercise group sessions were held weekly from week 2, either before or after the educational sessions on the weeks when educational sessions were also scheduled. *Braining* ^41^ consists of group exercise sessions at the psychiatric clinic, aiming for patients with mental disorders to increase their level of physical activity. The exercise sessions were from medium to vigorous intensity level, including aerobic and resistance training. The sessions lasted 30–45 min and were led by mental health staff at Psychiatry Southwest. Participation in a minimum of one group exercise session per week was required, but participants could attend additional sessions, which were offered several times a week.

Participants were offered three booster sessions approximately 1, 2 and 3 months post-intervention, via telephone/video or face to face, according to the participants’ choice, to check up on progress and help maintaining changes.

### Sample size

Because the aim of the study was to primarily evaluate the feasibility of the intervention, we did not aim for it to be powered to reveal statistically significant changes in the efficacy measures. We initially aimed to include 30 participants, divided in groups of six to ten participants, as this was considered sufficient to evaluate feasibility.

### Data analysis

Feasibility and acceptability measures were analysed with descriptive statistics. For the preliminary efficacy measures, intention-to-treat (ITT) analyses were performed, including all enrolled participants. To detect significant within-group changes from baseline to the 3-month follow-up (primary end-point), linear mixed-effects regression analyses for repeated measures with maximum likelihood estimation of parameters were implemented. Mixed-effects models use all available data, can properly account for correlation between repeated measurements on the same participant, have greater flexibility to model time effects and can handle missing data.^42^ For each preliminary efficacy measure, the model included fixed effects of time (baseline, post-intervention and 3-month follow-up). Participant effects were added as a random intercept factor to account for the variances between and within participants. Within-group effect sizes (Cohen’s *d*) were calculated. A McNemar test was used to measure change in the categorical variable tobacco use from baseline to the 3-month follow-up. All analyses were conducted in Stata version 16.1 for MacOS (StataCorp LLC). The statistical significance threshold was set at *P* < 0.05. Qualitative interviews were recorded and transcribed. The responses to the first set of questions of the qualitative interviews were summarised and are presented below.

## Results

### Participant flow and baseline characteristics

**Fig. 2:**
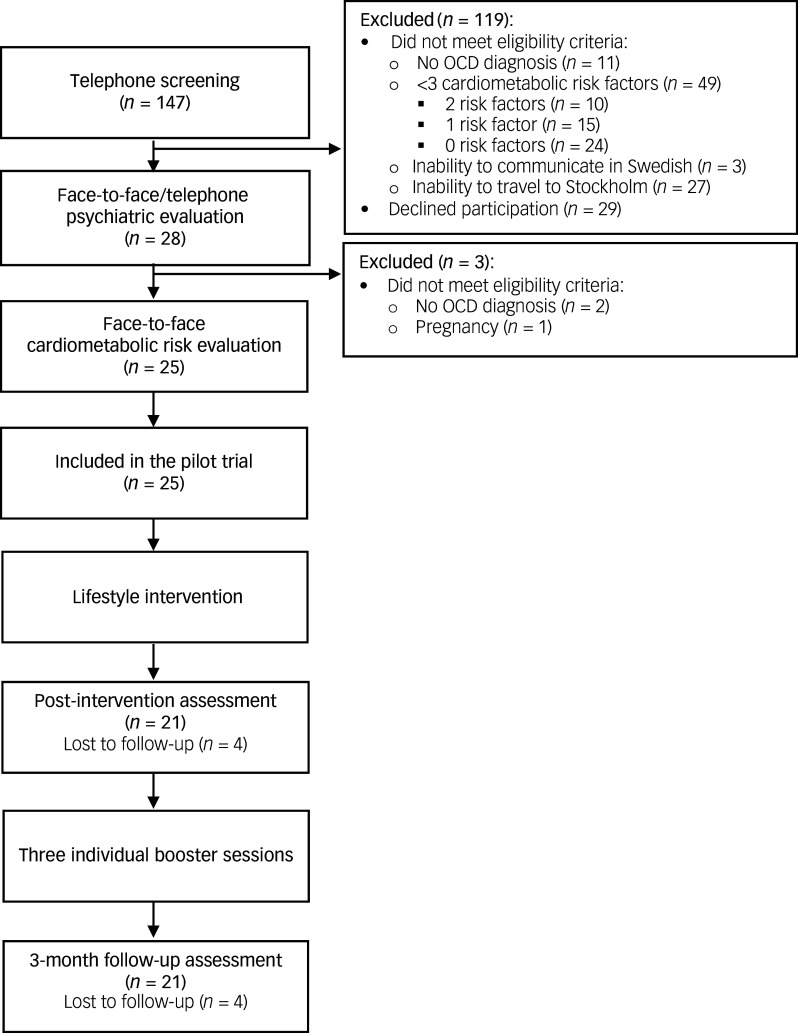
Participant flowchart. OCD, obsessive–compulsive disorder.

Figure 2 shows the participant flowchart. The 25 included participants were divided into four groups of six to seven participants each (Table 2). Most of the participants (*n* = 17, 68%) were women with a mean age of 37.44 years (s.d. = 10.90). About half of the sample (*n* = 13, 52%) were not working, either receiving disability pension, on sick leave, unemployed or in vocational rehabilitation (i.e. participating in supervised workplace activities after long-term unemployment or sick leave). Mean OCD severity at baseline, measured with the Y-BOCS, was 23.48 (s.d. = 5.99), corresponding to moderate OCD symptom severity.^43^ Sixteen (64%) individuals had a comorbid psychiatric condition and seven (28%) had a somatic condition at baseline. Most participants (*n* = 19, 76%) were on psychotropic medication and six (24%) were on medication for a cardiometabolic condition. Participants had a mean of 4.48 (s.d. = 1.23) cardiometabolic risk factors at baseline, out of the predefined list of 11 (Supplementary Table 2). The most common cardiovascular risk factors were being overweight and having abdominal obesity, both endorsed by 24 participants (96%).

**Table 2:** Demographic data and clinical characteristics of the sample (*N* = 25)

Variables	*n* (%)
Gender (women)	17 (68)
Age (years), mean (s.d.)	37.44 (10.90)
Age, minimum to maximum	21–60
Comorbidity	
Number of participants with psychiatric comorbidity	16 (64)
Autism spectrum disorder	8 (32)
Attention-deficit/hyperactivity disorder	4 (16)
Social anxiety disorder	4 (16)
Depression	3 (12)
Other psychiatric comorbidity	4 (16)
Number of participants with somatic comorbidity	7 (28)
Hypertension	5 (20)
Type 2 diabetes	2 (8)
Other somatic disorders	5 (20)
OCD symptom severity (Y-BOCS)	
Y-BOCS, mean (s.d.)	23.48 (5.99)
Mild	8 (32)
Moderate	12 (48)
Severe	5 (20)
Occupational status	
Disability pension/sick leave	8 (32)
Unemployment	3 (12)
Vocational rehabilitation	2 (8)
Student	4 (16)
Full-time/part-time employment	8 (32)
Treatment	
Ongoing cognitive–behavioural therapy	3 (12)
Ongoing medication for psychiatric disorder^a^	19 (76)
SSRI	14 (56)
Other psychotropic medication	10 (40)
Ongoing medication for cardiometabolic disorders^a^	6 (24)
Antihypertensives	5 (20)
Other cardiometabolic medication	3 (12)

OCD, obsessive–compulsive disorder; Y-BOCS, Yale–Brown Obsessive–Compulsive Scale; SSRI, selective serotonin re-uptake inhibitor.

a .Participants with more than one medication are listed in each corresponding row.

At baseline, self-reported physical activity was on average on a moderate level, whereas accelerometer data showed on average low daily physical activity (<5000 steps/day). The mean dietary index score was at the cut-off for an unhealthy diet (mean 4.08, s.e. = 0.42). Eight participants (32%) had risk alcohol consumption scores, as indicated by the AUDIT-C. They reported higher levels of stress, as measured by the PSS-10, than those described in the general population in Sweden (mean 24.92, s.e. = 1.48),^44^ and sleep problems above the cut-off on the ISI (mean 13.52, s.e. = 1.40)^45^ (Table 3). At the group level, weight, BMI, waist circumference, sagittal abdominal diameter and total and waist body fat exceeded the recommended values, whereas systolic and diastolic blood pressure, heart rate, all values from the blood samples and the Framingham risk score were in the normal range (Tables 4 and 5). However, high cholesterol levels and triglycerides were observed in three participants (12%), who were referred to primary care for further assessment. Additionally, one participant was referred to primary care because of a high 2-h glucose level (≥11.1 mmol/L) in the oral glucose tolerance test, indicating prediabetes or diabetes type 2. Finally, except for depressive symptoms, all mental health and quality of life measures were above the corresponding cut-offs at baseline, indicating clinically significant symptoms (Table 6).

**Table 3:** Model estimates for all lifestyle habits measures from baseline to the primary end-point (3-month follow-up) measurement from the linear mixed-effect models

Lifestyle habits	Mean (s.e.)^a^	Within-group difference		Within-group effect size
		coefficient (95% CI)^b^	*P*-value	Cohen’s *d* (95% CI)^c^
Physical activity (IPAQ MET^d^)				
Baseline	912.80 (323.05)			
Post-intervention	1464.93 (349.66)	552.13 (−233.68 to 1337.94)	0.168	0.34 (0.01–0.66)
3-month follow-up	1257.92 (365.18)	345.12 (−467.36 to 1157.60)	0.405	0.27 (−0.27 to 0.81)
Sedentary time (h)				
Baseline	9.07 (1.11)			
Post-intervention	7.50 (1.15)	−1.56 (−3.20 to 0.08)	0.062	−0.30 (−0.47 to −0.13)
3-month follow-up	9.32 (1.17)	0.25 (−1.45 to 1.95)	0.774	0.10 (−0.38 to 0.57)
Physical activity (steps per day)				
Baseline	4762.03 (678.62)			
Post-intervention	5074.64 (701.85)	312.61 (−588.93 to 1214.14)	0.497	0.11 (−0.17 to 0.40)
3-month follow-up	4660.90 (690.63)	336.21 (−530.86 to 1203.29)	0.447	0.10 (−0.22 to 0.43)
Dietary habits (Dietary index score)				
Baseline	4.08 (0.42)			
Post-intervention	5.23 (0.44)	1.15 (0.49–1.80)**	0.001	0.54 (0.23–0.85)
3-month follow-up	5.18 (0.44)	1.10 (0.45–1.75)**	0.001	0.52 (0.23–0.81)
Alcohol use (AUDIT-C)				
Baseline	2.84 (0.48)			
Post-intervention	2.24 (0.50)	−0.60 (−1.25 to 0.06)	0.073	−0.22 (−0.41 to −0.03)
3-month follow-up	1.96 (0.50)	−0.88 (−1.54 to −0.23)**	0.008	−0.35 (−0.64 to −0.06)
Stress (PSS-10)				
Baseline	24.92 (1.48)			
Post-intervention	21.19 (1.55)	−3.72 (−6.14 to −1.30)**	0.003	−0.49 (−0.89 to −0.10)
3-month follow-up	21.58 (1.55)	−3.34 (−5.76 to −0.92)**	0.007	−0.44 (−0.72 to −0.16)
Sleep (ISI)				
Baseline	13.52 (1.40)			
Post-intervention	11.79 (1.47)	−1.72 (−3.89 to 0.45)	0.120	0.23 (0.01–0.47)
3-month follow-up	11.76 (1.46)	−1.76 (−3.89 to 0.37)	0.106	−0.23 (−0.57 to 0.10)

IPAQ, International Physical Activity Questionnaire; MET, metabolic equivalent of task; AUDIT-C, Alcohol Use Disorders Identification Test-Concise; PSS-10, Perceived Stress Scale; ISI, Insomnia Severity Scale.

a .Estimated means and s.e. from the mixed-effects regression model.

b .Coefficients at the post-treatment and follow-up compare with the baseline time point.

c .Bootstrapped effect sizes (*d)* are derived from the mixed-effects regression model.

d .Responses on physical activity questions in IPAQ are converted to metabolic equivalent of task (MET)-min, according to a formula from the IPAQ scoring protocol.

**P<0.01.

**Table 4:** Model estimates for all physiological measures from baseline to the primary end-point (3-month follow-up) from the linear mixed-effect models

Physiological measures	Mean (s.e.)^a^	Within-group difference		Within-group effect size
		coefficient (95% CI)^b^	*P*-value	Cohen’s *d* (95% CI)^c^
Weight (kg)				
Baseline	95.64 (3.64)			
Post-intervention	94.73 (3.65)	−0.91 (−2.30 to 0.48)	0.198	−0.05 (−0.13 to 0.03)
3-month follow-up	96.09 (3.66)	0.45 (−0.93 to 1.84)	0.523	0.03 (−0.07 to 0.12)
Body mass index (kg/m^2^)				
Baseline	32.84 (1.11)			
Post-intervention	32.52 (1.11)	−0.31 (−0.78 to 0.16)	0.191	−0.06 (−0.14 to 0.03)
3-month follow-up	32.98 (1.11)	0.15 (−0.32 to 0.62)	0.545	0.03 (−0.08 to 0.13)
Waist circumference (cm)				
Baseline	108.46 (2.86)			
Post-intervention	106.77 (2.88)	−1.69 (−3.56 to 0.19)	0.078	−0.12 (−0.23 to -0.02)
3-month follow-up	107.20 (2.88)	−1.26 (−3.13 to 0.62)	0.189	−0.08 (−0.28 to 0.11)
Sagittal abdominal diameter (cm)				
Baseline	27.32 (0.86)			
Post-intervention	26.55 (0.87)	−0.77 (−1.45 to −0.08)*	0.029	−0.18 (−0.37 to 0.01)
3-month follow-up	27.55 (0.87)	0.23 (−0.45 to 0.92)	0.506	0.06 (−0.13 to 0.25)
Total body fat (%)				
Baseline	37.38 (1.57)			
Post-intervention	37.02 (1.58)	−0.37 (−1.40 to 0.67)	0.488	−0.04 (−0.16 to 0.07)
3-month follow-up	36.96 (1.58)	−0.43 (−1.46 to 0.60)	0.417	−0.05 (−0.18 to 0.08)
Fat around the waist (%)				
Baseline	35.16 (1.24)			
Post-intervention	35.62 (1.26)	0.46 (−0.69 to 1.60)	0.434	0.08 (−0.11 to 0.27)
3-month follow-up	36.61 (1.26)	1.45 (0.30−2.59)*	0.013	0.24 (0.07−0.40)
Systolic blood pressure (mmHg)				
Baseline	120.5 (2.25)			
Post-intervention	120.5 (2.39)	−0.02 (−4.37 to 4.33)	0.993	0.00 (−0.25 to 0.24)
3-month follow-up	118.7 (2.39)	−1.83 (−6.17 to 2.52)	0.409	−0.16 (−0.65 to 0.33)
Diastolic blood pressure (mmHg)				
Baseline	79.84 (1.23)			
Post-intervention	78.32 (1.31)	−1.52 (−4.03 to 0.99)	0.236	−0.24 (−0.57 to 0.10)
3-month follow-up	79.61 (1.31)	−0.23 (−2.75 to 2.28)	0.856	−0.05 (−0.47 to 0.37)
Resting heart rate				
Baseline	76.14 (2.37)			
Post-intervention	72.97 (2.53)	−3.17 (−8.22 to 1.88)	0.219	−0.32 (−0.72 to 0.07)
3-month follow-up	76.61 (2.53)	0.47 (−4.58 to 5.53)	0.854	0.02 (−0.41 to 0.46)
Framingham risk score^d^				
Baseline	3.77 (0.68)			
Post-intervention	3.44 (0.69)	−0.33 (−0.87 to 0.20)	0.220	−0.10 (−0.20 to 0.01)
3-month follow-up	3.39 (0.69)	−0.38 (−0.93 to 0.16)	0.167	−0.11 (−0.34 to 0.12)

a .Estimated means and s.e. from the mixed-effects regression model.

b .Coefficients at the post-treatment and follow-up compare with the baseline time point.

c .Bootstrapped effect sizes (*d)* are derived from the mixed-effects regression model.

d .Calculated based on the gender assigned at birth.

* *P* < 0.05.

**Table 5:** Model estimates for all laboratory measures from baseline to the primary end-point (3-month follow-up) from the linear mixed-effect models

Laboratory measures	Mean (s.e.)^a^	Within-group difference		Within-group effect size
		coefficient (95% CI)^b^	*P*-value	Cohen’s *d* (95% CI)^c^
Total cholesterol (mmol/L)				
Baseline	4.83 (0.21)			
Post-intervention	4.71 (0.22)	−0.12 (−0.40 to 0.17)	0.422	−0.11 (−0.40 to 0.18)
3-month follow-up	4.94 (0.22)	0.11 (−0.18 to 0.41)	0.450	0.13 (−0.23 to 0.50)
LDL cholesterol (mmol/L)				
Baseline	2.96 (0.18)			
Post-intervention	2.88 (0.18)	−0.09 (−0.31 to 0.14)	0.456	−0.10 (−0.37 to 0.18)
3-month follow-up	2.97 (0.19)	0.01 (−0.22 to 0.24)	0.938	0.03 (−0.31 to 0.36)
HDL cholesterol (mmol/L)				
Baseline	1.32 (0.07)			
Post-intervention	1.31 (0.07)	−0.002 (−0.10 to 0.09)	0.960	−0.01 (−0.27 to 0.25)
3-month follow-up	1.29 (0.08)	−0.02 (−0.12 to 0.07)	0.648	−0.05 (−0.33 to 0.23)
Triglycerides (mmol/L)				
Baseline	1.19 (0.16)			
Post-intervention	1.19 (0.16)	−0.01 (−0.20 to 0.18)	0.926	−0.01 (−0.13 to 0.10)
3-month follow-up	1.48 (0.16)	0.28 (0.09−0.47)**	0.004	0.35 (0.09−0.61)
Glucose (mmol/L)				
Baseline	5.32 (0.09)			
Post-intervention	5.46 (0.10)	0.14 (−0.04 to 0.33)	0.128	0.33 (−0.02 to 0.69)
3-month follow-up	5.59 (0.10)	0.27 (0.08−0.45)**	0.005	0.56 (0.23−0.89)
HbA1c (mmol/L)				
Baseline	34.48 (0.72)			
Post-intervention	35.19 (0.74)	0.71 (0.01−1.42)*	0.048	0.20 (0.06−0.34)
3-month follow-up	34.71 (0.74)	0.23 (−0.49 to 0.95)	0.528	0.06 (−0.13 to 0.25)
C-reactive protein				
Baseline	4.67 (1.12)			
Post-intervention	4.91 (1.17)	0.23 (−1.58 to 2.05)	0.801	0.02 (−0.16 to 0.20)
3-month follow-up	3.36 (1.18)	−1.32 (−3.16 to 0.53)	0.162	−0.23 (−0.66 to 0.20)
2h oral glucose tolerance test				
Baseline	5.14 (0.31)			
3-month follow-up	5.59 (0.41)	0.45 (−0.47 to 1.37)	0.340	0.18 (−0.21 to 0.56)

LDL, low-density lipoprotein; HDL, high-density lipoprotein; HbA1c, glycated haemoglobin.

a .Estimated means and s.e. from the mixed-effects regression model.

b .Coefficients at the post-treatment and follow-up compare with the baseline time point.

c .Bootstrapped effect sizes (*d)* are derived from the mixed-effects regression model.

*P<0.05; **P<0.01.

**Table 6:** Model estimates for all psychometric measures from baseline to the primary end-point (3-month follow-up) measurement from the linear mixed-effect models

Psychometric measure	Mean (s.e.)^a^	Within-group difference	*P*-value	Within-group effect size
		coefficient (95% CI)^b^		Cohen’s *d* (95% CI)^c^
Y-BOCS				
Baseline	23.48 (1.23)			
Post-intervention	19.17 (1.28)	−4.31 (−6.14 to −2.48)***	<0.001	−0.71 (−0.95 to −0.48)
3-month follow-up	19.79 (1.28)	−3.69 (−5.52 to −1.86)***	<0.001	−0.56 (−0.85 to −0.26)
OCI-12				
Baseline	24.84 (2.00)			
Post-intervention	21.94 (2.05)	−2.90 (−5.17 to −0.62)*	0.013	−0.29 (−0.54 to −0.03)
3-month follow-up	23.33 (2.05)	−1.51 (−3.79 to 0.76)	0.192	−0.15 (−0.33 to 0.03)
PHQ-9				
Baseline	12.92 (1.27)			
Post-intervention	10.35 (1.31)	−2.56 (−4.35 to −0.79)**	0.005	−0.40 (−0.66 to −0.14)
3-month follow-up	11.16 (1.31)	−1.76 (−3.54 to 0.02)	0.053	−0.26 (−0.50 to −0.02)
WSAS				
Baseline	20.48 (2.22)			
Post-intervention	19.47 (2.26)	−1.01 (−3.15 to 1.13)	0.356	−0.09 (−0.26 to 0.09)
3-month follow-up	17.28 (2.26)	−3.20 (−5.34 to −1.06)**	0.003	−0.27 (−0.46 to −0.09)
EQ-5D-3L index score				
Baseline	0.43 (0.06)			
Post-intervention	0.61 (0.06)	0.17 (0.08−0.27)**	0.001	0.62 (0.29−0.95)
3-month follow-up	0.53 (0.06)	0.09 (−0.01 to 0.19)	0.064	0.30 (−0.01 to 0.62)
EQ-5D-3L visual analogue scale				
Baseline	46.28 (3.79)			
Post-intervention	53.86 (4.02)	7.58 (0.53−14.63)*	0.035	0.38 (0.01−0.75)
3-month follow-up	51.20 (4.02)	4.92 (−2.13 to 11.97)	0.172	0.24 (−0.17 to 0.64)

Y-BOCS, Yale−Brown Obsessive−Compulsive Scale; OCI-12, Obsessive−Compulsive Inventory; PHQ-9, Patient Health Questionnaire-9; WSAS, Work and Social Adjustment Scale; EQ-5D-3L, EuroQol Five-Dimensional Three-Level Questionnaire.

a .Estimated means and s.e. from the mixed-effects regression model.

b .Coefficients at the post-treatment and follow-up compare with the baseline time point.

c .Bootstrapped effect sizes (*d)* are derived from the mixed-effects regression model.

*P < 0.05; **P < 0.01; ***P < 0.001.

### Aim 1: feasibility and acceptability

A total of 147 participants were screened to include 25 participants (Fig. 2). Participants were recruited between November 2022 and November 2023, with the last follow-up data collected in May 2024. Recruitment was only active during the approximately 2-month period before a group started and then paused until the next group was planned to start. During this period, we were able to recruit 25 of the planned 30 participants. Recruitment was terminated when the fourth group was filled. Most of the exclusions after the initial screening were because participants did not fulfil the criteria of at least three cardiometabolic risk factors (*n* = 49). Reasons for declining participation (*n* = 29) at this stage primarily included difficulties joining sessions because of work. Initially, the main source of recruitment was the clinic, but recruitment pace increased when we advertised through the OCD patient organisation and social media. Most participants were self-referrals (*n* = 20, 80%), and the rest came from a psychiatric clinic, primarily the specialist OCD clinic (*OCD-programmet*).

Adherence varied among participants. On average, participants joined 3.60 (s.d. = 2.30) of the six educational sessions (60%) and 5.60 (s.d. = 4.10) of the 12 exercise sessions (47%) (Supplementary Fig. 1). The main reported reasons for not attending included illness and unavailability owing to work or studies.

Full results for the intervention credibility questionnaire and the CSQ-8, used to measure intervention satisfaction, are presented in Supplementary Table 3. Overall, the intervention was rated as credible. Participants considered the intervention well suited for individuals with OCD to change their lifestyle habits (mean 3.04, s.d. = 0.89; on a scale of 0–4), and they reported to be motivated to participate (mean 3.36, s.d. = 0.95, on a scale of 0–4). They scored lower on how much they thought the intervention would help them change their lifestyle habits (mean 2.80, s.d. = 0.87, on a scale of 0–4). Intervention satisfaction was high (CSQ-8: mean 26.40, s.d. = 4.30).

As for the attrition rates, four (16%) participants, from three of the four groups, ended participation prematurely and did not provide further data beyond the baseline assessment. The reasons for dropping out included worsening of the OCD symptoms, pregnancy, health problems, and moving out of Stockholm and therefore being unable to attend. Data on all three time points were then collected from 21 participants (84%).

There were three severe adverse events. One participant was admitted to hospital and one had to seek emergency care because of somatic conditions during the lifestyle intervention period, and another attempted suicide that required specialist emergency care during the follow-up. These events were all assessed as unrelated to the intervention. Results from the self-reported adverse events questionnaire are presented in Supplementary Table 4. Overall, participants scored low on all items (mean < 2, on a scale of 0–4).

### Aim 2: preliminary efficacy

Estimated means and s.e. from the linear mixed-effects regression analyses are shown in Tables 3– 6. Raw means and s.d. are presented in Supplementary Table 5.

#### Lifestyle habits

Participants showed an improvement from baseline to the 3-month follow-up in dietary habits (dietary index score coefficient 1.10, 95% CI 0.45−1.75; *P* = 0.001); a reduction in alcohol consumption, as measured with the AUDIT-C (−0.88, 95% CI −1.54 to −0.23; *P* = 0.008); and an improvement in perceived stress scores, as measured with the PSS-10 (−3.34, 95% CI −5.76 to −0.92; *P* = 0.007) (Table 3). The within group-effect sizes (Cohen’s *d,* 95% CI) were small to medium (dietary index score: *d* = 0.52, 95% CI 0.23−0.81; AUDIT-C: *d* = −0.35, 95% CI −0.64 to −0.06; PSS-10: *d* = −0.44, 95% CI −0.72 to −0.16). No significant changes were found in physical activity and sedentary behaviour (IPAQ) and sleep (ISI). Of the six participants who used tobacco at baseline, one stopped smoking by the end of the intervention, which was maintained at the 3-month follow-up. This was not significant in the McNemar test (*χ* ^2^(1) = 1.00, *P* = 0.317), based on 21 completers.

#### Anthropometric measurements and blood samples

Changes on these variables are shown in Tables 4 and 5. There was a slight reduction on sagittal abdominal diameter from baseline to post-intervention (−0.77, 95% CI −1.45 to −0.08; *P* = 0.029), but at the primary end-point this change was no longer statistically significant. HbA1c was significantly higher at post-intervention (0.71, 95% CI 0.01−1.42; *P* = 0.048), but not at the primary end-point. Fat around the waist, fasting triglycerides and fasting glucose levels were significantly higher at follow-up, compared with baseline (fat around the waist: 1.45, 95% CI 0.30−2.59, *P* = 0.013; fasting triglycerides: 0.28, 95% CI 0.09−0.47, *P* = 0.004; fasting glucose: 0.27, 95% CI 0.08−0.45, *P* = 0.005). The within-group effect sizes were small to medium (fat around the waist: *d* = 0.24, 95% CI 0.07−0.40; fasting triglycerides: *d* = 0.35, 95% CI 0.09−0.61; fasting glucose: *d* = 0.56, 95% CI 0.23−0.89).

#### Mental health and quality of life

Participants showed a statistically significant reduction from baseline to the primary end-point in clinician-rated OCD symptom severity (Y-BOCS: −3.69, 95% CI −5.52 to −1.86; *P* < 0.001), corresponding to a moderate effect (*d* = −0.56, 95% CI −0.85 to −0.26). A statistically significant improvement in functioning was observed (WSAS: −3.20, 95% CI −5.34 to 1.06; *P* = 0.003) (*d* = −0.27, 95% CI −0.46 to −0.09). Self-reported OCD symptoms, depressive symptoms and quality of life were significantly improved from baseline to the post-intervention assessment, but not to the 3-month follow-up (Table 6).

### Aim 3: participants’ experiences

Of the 21 completers, 17 agreed to be interviewed. Overall, participants were satisfied with the intervention. Particularly, participants valued getting practical lifestyle advice, taking part in guided exercise sessions in a supportive environment and being in a group where all had OCD. Most participants (*n* = 15) reported having made some changes in their lifestyle, primarily increasing physical activity and changing dietary habits. Participants also provided feedback to improve the intervention; they expressed that the lifestyle intervention was too short, that they would have wanted more sessions over a longer period and more room for discussion and interaction during group sessions. Some of the participants who were employed expressed difficulties attending because of work, and wished for sessions outside of working hours.

## Discussion

We developed a lifestyle intervention, called LIFT, to improve lifestyle habits and reduce cardiometabolic risk factors, and tested it in 25 individuals with OCD in a feasibility trial. To our knowledge, this is the first lifestyle intervention specifically tailored to individuals with OCD and increased cardiometabolic risk. The intervention included OCD-specific content and adaptations, and combined both educational sessions on healthy lifestyle habits and exercise sessions to increase physical activity.

Our study participants rated the intervention as credible and reported being satisfied. The intervention can be described as generally safe, with most reported adverse events being not severe and considered unrelated to the intervention. However, the recruitment rate was lower than expected, with 147 individuals screened to include 25 participants. Given the relative difficulties recruiting, we decided to stop recruitment before reaching our initial goal of 30 participants. The main reason for exclusion was that individuals did not have the three cardiometabolic risk factors required for inclusion. Other lifestyle intervention trials with higher recruitment rates have often targeted only one specific risk factor (e.g. overweight or hypertension),^16,18^ whereas our participants needed to have at least three, making recruitment more challenging. Although we wanted to ensure that we targeted individuals that were in need of help, we may have missed individuals that would have benefited from participating in the intervention but had fewer risk factors. The fact that over 100 individuals showed interest in the study is indicative of this population being motivated to change their lifestyle habits, as previously reported in this group.^11^ Although retention rate was high (84%), a few participants were not very engaged in the intervention and only attended some of the sessions, which could have affected the results, as previous studies have found associations between a higher attendance to lifestyle intervention sessions and weight loss.^16,17^ On average, participants attended 60% of the group sessions and 47% of the exercise sessions. Despite the differences in design, the attendance to the educational group sessions was similar to the 60.2% reported in the STRIDE study, an RCT evaluating a lifestyle intervention for individuals on antipsychotic medication.^16^ However, both our own trial and STRIDE showed lower attendance than the PREMIER trial (approximately 80%), which targeted a non-psychiatric population.^13^ Individuals with psychiatric conditions may find it more difficult to adhere to these programmes and may require additional support to remain engaged. Previous trials testing exercise-based interventions in OCD have shown higher adherence rates (>80%), but these studies were small and a monetary incentive system was used to improve adherence.^19^ Moreover, these interventions were aimed at reducing OCD symptom severity and not cardiometabolic risk factors, which may have affected motivation.

Our second aim was to test the preliminary efficacy of LIFT at changing lifestyle habits, physiological measures and mental health variables. We found significant improvements in dietary habits, alcohol consumption, perceived stress, clinician-reported OCD symptom severity, and functioning. Positive effects on lifestyle habits, primarily increased vegetable intake and increased physical activity, have been found in other lifestyle interventions in individuals with mental disorders.^15^ In our sample, no significant improvements in physical activity, sleep and anthropometric or laboratory measures were found. Many of these values were in the normal range already at baseline, limiting room for improvement. That, together with the fact that the sample was not powered to detect changes in these variables, as this was not the primary aim, may explain the lack of significant changes. Additionally, effect sizes in physical activity interventions are often small, even if changes are clinically significant. A meta-synthesis reported a median effect size across trials of *d* = 0.20,^46^ similar to our self-reported physical activity results. It has further been shown that any increase in physical activity can lead to important health benefits, especially for the most inactive.^47^ Nonetheless, an additional explanation could be that the intervention, as currently designed, is not sufficient to promote and maintain changes. This should be tested in a properly powered RCT.

Our final aim was to collect feedback on the participants’ experience of participation in LIFT to help improving both the intervention and the procedures of the study. Overall, the participants expressed that they were satisfied with the intervention and that they had started to make some lifestyle changes. However, many wished for a longer and/or more intense intervention. Further, some participants found it difficult to join sessions because of work. This was also a reason for some individuals declining participation in the recruitment phase.

Overall, LIFT was generally feasible for adults with OCD and cardiometabolic risk. These results are very promising because prevention strategies, including lifestyle interventions, together with better surveillance and early intervention strategies, have been suggested to avoid fatal outcomes in OCD.^2^ Nonetheless, the efficacy of this intervention needs to be tested in a fully powered RCT. Future trials should consider whether the target population should be changed to improve recruitment rates. Inclusion criteria could solely focus on behavioural risk factors (i.e. unhealthy lifestyle habits), regardless of the presence of physiological measures of cardiometabolic risk, as this has been suggested as a sufficient reason to intervene.^48^ Additionally, there are several ways in which the intervention could be improved. Adherence may be increased by offering sessions at different times to better suit participants who are employed. Also, it was noted that attendance to the weekly exercise group sessions was higher on weeks with educational group sessions, which were held every other week, indicating that a different session planning may further improve adherence. Results from the preliminary efficacy measures, together with the participants’ feedback, suggest that they may benefit from a longer intervention and more support to promote and maintain changes.

This study has several limitations. Given the small sample size and lack of control group, any preliminary efficacy results should be interpreted with caution. Some participants were receiving CBT and/or psychiatric medication during their participation and, without a control group, we cannot rule out that any observed improvements could be attributed to these concomitant interventions. The 3-month follow-up time point may have been too short for some of the preliminary efficacy outcomes to change. Future trials should consider longer follow-ups. Additionally, most participants self-referred to the study, which may have led to a particularly motivated sample, limiting the generalisability of the results. Further, many of our outcomes were self-reported. Previous studies have found that self-reported physical activity and dietary habits often are inaccurate.^49,50^ To tackle this, accelerometers were used as an objective measure of physical activity. However, accelerometers do not register some activities (e.g. swimming) and, since the accelerometer is taken off during the night, some participants reported that they forgot to put it back on, resulting in data uncertainties. Additionally, dietary habits were measured with a short screening tool widely used in Sweden, chosen to reduce participant burden. However, the questionnaire does not take into account, for example, energy intake. Finally, our participants were relatively young, and some outcome measures used in the trial, such as the Framingham risk score, are less likely to classify younger individuals as being high risk.^51^

In summary, LIFT is an overall feasible lifestyle intervention for adults with OCD who have elevated cardiometabolic risk. The biggest challenges to this study were recruitment and the modest adherence to the intervention. The preliminary efficacy results for changes in lifestyle habits, mental health, and functioning are promising, but must be confirmed in larger samples using a control group. Lessons learned in this study will inform the design of a future RCT, including changes to the inclusion criteria and the intervention itself.

## Supporting information

Holmberg et al. supplementary material 1 Holmberg et al. supplementary material

Holmberg et al. supplementary material 2 Holmberg et al. supplementary material

## Data Availability

The data that support the findings of this study are not publicly available due to individual privacy of the participants, but could be made available to other researchers from the corresponding author, A.H., on reasonable request.

## Supplementary material

The supplementary material is available online at https://doi.org/10.1192/bjo.2025.10774
