# Supplementary material for: A structured lifestyle intervention to reduce cardiometabolic risk factors in individuals with obsessive–compulsive disorder: feasibility trial
Source: BJPsych Open. 2025 Aug 1;11(5):e171. doi: 10.1192/bjo.2025.10774 (PMC12344427; doi:10.1192/bjo.2025.10774)
Supplement: Holmberg et al. supplementary material 1 — Holmberg et al. supplementary material [file S2056472425107746sup001.docx]

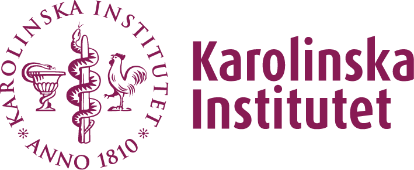


**Protocol Lifestyle Intervention for OCD**

| Long title of the trial | A structured lifestyle intervention to reduce cardiometabolic risk factors in individuals with obsessive-compulsive disorder – A feasibility study |
| --- | --- |
| Short title of trial | Lifestyle intervention for OCD |
| Version and date of protocol | Version 1.4, 2024-02-14 |
| Sponsor | Psykiatri Sydväst, Stockholms läns sjukvårdsområde (SLSO), Region Stockholm |
| Funder (s) | FORTE (grant number 2019-00438), the Swedish Research Council (grant number 2022-005109), Region Stockholm / ALF Medicin project grant (grant number 20200139), Hjärt-Lungfonden (grant numbers 20210493 and 20220899), and Committee for Research at Karolinska Institutet (grant numbers 2020-01361 and 2022-01675) |
| Trial registration | Open Science Framework: <https://osf.io/wmxbp> |
| Ethical Review | Swedish Ethical Review Authority approval number: 2022-00375-01  Amendments: 2022-03546-02, 2023-01962-02, 2024-00708-02 |
| Sites | Department of Cardiology, Karolinska Universitetssjukhuset Solna; OCD-programmet, Karolinska Universitetssjukhuset Huddinge |
| Principal investigator | Dr Lorena Fernández de la Cruz  Department of Clinical Neuroscience (CNS), K8  CPF Mataix-Cols |
| Sponsor representative | Dr Lina Martinsson  Psykiatri Sydväst, Stockholm Healthcare Services, Huddinge sjukhusområde |

**VERSION HISTORY**

| **Version number** | **Version date** | **Reasons for change** |
| --- | --- | --- |
| 1.0 | 2022-01-18 | First version of the study protocol. Submitted to the Swedish Ethical Review Authority. |
| 1.1 | 2022-03-25 | - In section 6.2, we have removed that participants will be encouraged to go the gym or exercise at home, after request from the Swedish Ethical Review Authority, due to insurance-related concerns. - In section 7.2, Cardiometabolic risk, outcome 14), it has been clarified that we are collecting samples on genetic biomarkers. White blood cell telomere length was added to this sentence instead of stated separately as in version 1.0. A corresponding change has been done in Table 2. |
| 1.2 | 2022-06-08 | - We have removed one risk factor from the list of cardiometabolic risk factors in the inclusion criteria: “**Insulin resistance:** Confirmed by the study nurse at the initial cardiometabolic evaluation, after analyzing blood sample collected after >8 h fast, and calculating the Homeostatis Model Assessment Index for Insulin Resistance (HOMA-IR): fasting glucose [mmol/L] × fasting insulin [μU/mL]/22.5. Common reference levels for HOMA-IR insulin resistance range from 0.7 - 2.0.”   The reason for removing it is that it requires a complicated procedure and it is deemed that we would get sufficient information on this variable from the oral glucose tolerance test.   - The 6-month follow-up has been removed and, instead, measures will only be collected at baseline, post-intervention, and at the 3-month follow-up. An amendment has been submitted to the ethical committee to approve this change.   The changes in this version of the protocol have been done before the start of the recruitment. |
| 1.3 | 2022-12-21 | - The oral glucose tolerance test at post-intervention has been removed since glucose levels take longer than 3 months to change, the test takes long time, and it can be perceived as too demanding by the participants. The measures are kept at baseline and at the 3-month follow-up. - We have amended an error in Table 2: Fasting insulin was removed since this was not a planned outcome measure. - In section 9 regarding statistical analysis, it has been clarified that the linear mixed effects regression analyses were for baseline to post-assessment and baseline to the 3-month follow up. It has been added that results would be stratified by gender, power allowing. - Ethical amendments with reference numbers 2022-03546-02, approved 2022-07-11 (referring to change in data collection time points) have been added to page 1. |
| 1.4 | 2024-02-14 | - The list of funders in the cover page of the protocol and in section 14 about the financing sources has been updated. - Ethical amendment with reference number 2023-01962-02, approved 2023-04-06 (referring to prolonging the period to store biobank blood samples) have been added to page 1. - Additional questions have been added to the interview guide (part 2). The questions are now part of the Appendix and this additional set of questions is also described in section 7.4. An ethical amendment with reference number 2024-00708-02 regarding this addition has been approved on 2024-02-14, and added to page 1. |

**SIGNATURE**

Protocol version number: 1.4

Lorena Fernández de la Cruz, Principal Investigator

Date: 14^th^ February 2024


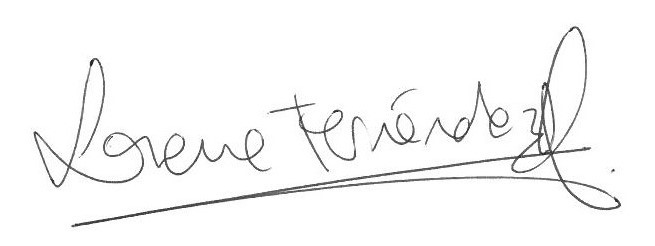


Signature:

# TRIAL PERSONNEL

Principal Investigator: Dr Lorena Fernández de la Cruz, Karolinska Institutet, Department of Clinical Neuroscience (CNS), K8, Gävlegatan 22, Plan 8 113 30 Stockholm, Sweden [lorena.fernandez.de.la.cruz@ki.se](mailto:lorena.fernandez.de.la.cruz@ki.se)

Sponsor representative: Dr Lina Martinsson, Psykiatri Sydväst, Stockholm Healthcare Services, Huddinge sjukhusområde M58, Medicingatan 8, plan 5, 141 86, Stockholm, Sweden [lina.martinsson@ki.se](mailto:lina.martinsson@ki.se)

Co-investigators: Professor David Mataix-Cols, Karolinska Institutet, Department of Clinical Neuroscience (CNS), K8, Gävlegatan 22, Plan 8 113 30 Stockholm, Sweden [david.mataix.cols@ki.se](mailto:david.mataix.cols@ki.se)

Professor Christian Rück, Karolinska Institutet, Department of Clinical Neuroscience (CNS), K8, Psykiatri Sydväst, Stockholm Healthcare Services, Huddinge sjukhusområde M46, Medicingatan 8, plan 4, 141 86, Stockholm, Sweden [christian.ruck@ki.se](mailto:christian.ruck@ki.se)

Dr Matthias Lidin, Norrbacka S1:02, Karolinska Universitetssjukhuset Solna, 17176 Stockholm, Sweden

[matthias.lidin@ki.se](mailto:matthias.lidin@ki.se)

Dr Kayoko Isomura, Karolinska Institutet, Department of Clinical Neuroscience (CNS), K8, Gävlegatan 22, Plan 8 113 30 Stockholm, Sweden [kayoko.isomura@ki.se](mailto:kayoko.isomura@ki.se)

Professor Matteo Bottai, Karolinska Institutet, Insitute of Environmental Medicine, C6, Nobels väg 13, 17177 Stockholm, Sweden [matteo.bottai@ki.se](mailto:matteo.bottai@ki.se)

Anna Holmberg, Karolinska Institutet, Department of Clinical Neuroscience (CNS), K8, Gävlegatan 22, Plan 8 113 30 Stockholm, Sweden [anna.holmberg.2@ki.se](mailto:anna.holmberg.2@ki.se)

# SUMMARY

| **Title:** | A structured lifestyle intervention to reduce cardiometabolic risk factors in individuals with obsessive-compulsive disorder – a feasibility study |
| --- | --- |
| **Short title:** | Lifestyle intervention for OCD |
| **Objectives:** | The overall aim of this study is to evaluate the feasibility of a lifestyle intervention to reduce cardiometabolic risk factors in individuals with OCD. Specific aims include to: 1) evaluate the acceptability of the intervention, 2) evaluate its preliminary efficacy, and 3) systematically gather participants’ perspectives and responses to different components of the intervention and how OCD symptoms affect lifestyle habits, the ability to make lifestyle changes, as well as experiences of seeking and receiving somatic health care, in order to further improve the intervention before conducting a large-scale, fully-powered efficacy trial. |
| **Type of trial:** | Single-group feasibility trial |
| **Trial design and methods:** | All potential participants are initially screened via the telephone. This is followed by an inclusion assessment which includes a face-to-face/phone psychiatric interview and an in-person medical assessment to evaluate cardiometabolic variables. Participants who are eligible and have consented to participate will be offered to take part in the intervention which will include 1 individual session with a clinical psychologist and a nurse + 6 psychoeducation group sessions on lifestyle habits + 12 exercise sessions.  Participants will complete measures at baseline, post-intervention, and 3 months post-intervention.  The primary outcome measures are ease of recruitment, participant retention, adherence to the intervention, credibility, safety, and satisfaction with the intervention at post-intervention, which will constitute measures of intervention feasibility and acceptability. Secondary outcome measures include changes in lifestyle habits, cardiometabolic physiological measurements, OCD severity, functional impairment, and quality of life measured at post and 3 months after the end of the intervention. |
| **Rationale for study:** | OCD is a prevalent and impairing disorder. Mortality rates in individuals with OCD are higher than in the general population, as is the risk of metabolic and cardiovascular diseases. These risks remain significant over and above psychiatric comorbidities and shared familial factors, indicating that at least part of this risk could be a consequence of the lifestyles of these individuals (e.g., physical inactivity, unhealthy diet). However, no lifestyle interventions targeting cardiometabolic risk factors for people with OCD have ever been evaluated.  Prior to a fully-powered randomized controlled trial evaluating the efficacy and cost-effectiveness of an intervention to reduce cardiometabolic risk factors in individuals with OCD, a feasibility trial is needed to establish the acceptability and credibility of the intervention, participants’ satisfaction, and potential adverse events, as well as to provide preliminary efficacy data. |
| **Participant time in trial:** | Approximately 6 months. |
| **Total trial duration:** | Approximately 18 months (from enrolment of first participant to last participant’s measurement point). |
| **Planned trial sites:** | Psychiatric assessments will be administered face-to-face at Karolinska Universitetssjukhuset, Solna or on the phone. Physiological assessments will be in-person also at Karolinska Universitetssjukhuset, Solna. The intervention will be held at Karolinska Universitetssjukhuset, Huddinge. Since both the assessments and the intervention require in-person participation, the recruitment will be restricted to the Stockholm Region. |
| **Sample:** | 30 adults with OCD and at least three known cardiometabolic risk factors. |
| **Brief eligibility criteria:** | Eligible participants will be aged 18 years or older and have a DSM-5 diagnosis of OCD and at least 3 cardiometabolic risk factors, out of a predefined list. Exclusion criteria will include language limitations, inability to travel to Stockholm, inability to attend the intervention sessions, intellectual disability or acute psychiatric symptoms or suicidal risk that could interfere with the intervention, being pregnant or breastfeeding, having had a myocardial infarction or stroke within the previous 6 months, cardiometabolic risk measures much over the normal range or initiation or adjustment of any cardiometabolic medication within 3 months prior to assessments. |
| **Statistical analysis:** | *Demographic and clinical data at baseline* will be summarised using descriptive statistics.  *Feasibility and acceptability of the intervention:* Descriptive statistics on ease of recruitment, participant retention, adherence to the intervention, credibility, safety, and satisfaction.  *Preliminary efficacy*: Measures of lifestyle habits, cardiometabolic risk, OCD severity, functional impairment, and quality of life will be analysed through linear mixed models to detect within group change over time. |

# INTRODUCTION

Obsessive-compulsive disorder (OCD) is characterized by obsessions (recurrent and intrusive thoughts, urges or images that cause anxiety) and compulsions (ritualistic behaviors or mental acts that the individual feels an urge to repeat over and over to reduce the distress caused by the obsessions) (American Psychiatric Association, 2013). OCD is one of the most common psychiatric disorders, affecting approximately 2% of the population (Ruscio et al., 2008). It is a disabling disorder and, if untreated, can become chronic. There are well-validated evidence-based treatments for OCD, including cognitive behavioral therapy (CBT) and serotonin reuptake inhibitors (SRIs) (American Psychiatric Association, 2007; NICE, 2006).

Mortality rates in individuals with OCD have shown to be higher than in the general population (Meier et al., 2016), which may be partially explained by a higher risk of dying by suicide(Fernandez de la Cruz et al., 2017; Meier et al., 2016), although it is also likely that the increased premature mortality is related to natural causes (i.e.,, higher rates of health-related problems). Previous investigations by our team have shown a 45% increased risk for cardiovascular and metabolic disorders in OCD, compared to the general population. The risks were the highest for obesity, a broadly-defined group of circulatory system diseases, and type 2 diabetes mellitus (Isomura et al., 2018). Further, individuals with OCD, compared to matched controls without the disorder, have shown to have a moderately higher risk of a broad range of cardiovascular diseases, with the strongest associations for the subtypes venous thrombo-embolism and heart failure (Isomura et al., 2021). In both these studies, the risks remained increased over and above psychiatric comorbidities and shared familial factors. Hence, it is likely that at least part of these described risks are a consequence of the OCD itself and the lifestyles of the individuals with the disorder (e.g., physical inactivity, unhealthy diet).

Cardiovascular diseases are the number one cause of death globally, and can be prevented by addressing risk factors such as tobacco use, an unhealthy diet, obesity, physical inactivity, and harmful use of alcohol (World Health Organization, 2017). Several interventions to change unhealthy lifestyle habits that can contribute to the risk of cardiometabolic disorders have previously been evaluated in the general population. Results indicate that targeting lifestyle factors can lead to changes in indicators of cardiometabolic risk, such as high blood pressure, obesity, and total cholesterol (Lidin et al., 2018; Svetkey et al., 2003). There is also an increasing number of studies targeting lifestyle factors in populations with psychiatric disorders, but these are mainly focused on people with bipolar disorder and psychotic disorders, such as schizophrenia (Cabassa et al., 2010; Green et al., 2015). Results show that these interventions, including for example implementation of a healthy diet and an increase of physical activity, lead to health improvements in the cardiometabolic profile of these patients (Cabassa et al., 2010; Green et al., 2015). However, to our knowledge, no lifestyle interventions targeting cardiometabolic risk factors for people with OCD have ever been evaluated. A few pilot investigations have tested exercise as an adjunct intervention to CBT in order to reduce OCD symptoms, but they were based on small samples (range 11 to 56 participants) (Abrantes et al., 2017; Brown et al., 2007; Rector et al., 2015). In one of these studies, Abrantes et al. (2017) conducted a pilot randomized controlled trial (RCT), including 56 participants, that tested the effect of an aerobic exercise group (n=28) or a health education group (n=28) on OCD symptoms. In this study, cardiorespiratory fitness (maximum oxygen uptake, VO_2_) was one secondary outcome measure and, although the sample was small and underpowered to detect between-group significant differences, results showed a significant increase in VO_2_ in the group on aerobic exercise (i.e., better cardiorespiratory fitness). This indicates that lifestyle interventions can be feasible and acceptable for individuals with OCD.

# Objectives

The overall aim of this study is to evaluate the feasibility of a lifestyle intervention to reduce cardiometabolic risk factors in individuals with OCD. Specific aims are listed below:

1. To establish the feasibility and acceptability of the developed intervention, by measuring ease of recruitment, participant retention, adherence, credibility, safety, and satisfaction, measured at post-intervention.
2. To test the preliminary efficacy of this intervention (e.g., changes in lifestyle habits, cardiometabolic physiological and laboratory measurements, OCD severity, functional impairment, and quality of life) 3 months after the intervention. Follow-up measures of these variables will also be collected at post-treatment.
3. To explore participants’ perspectives and responses to different components of the intervention and how OCD symptoms affect lifestyle habits, the ability to make lifestyle changes, as well as experiences of seeking and receiving somatic health care, by conducting semi-structured qualitative interviews, which will be used to improve the initially developed and tested intervention.

# Project description

## Design

This feasibility study will use an open trial design with no control group. Quantitative and qualitative data on the feasibility, acceptability, and preliminary efficacy of the lifestyle intervention will be gathered.

## Study sites

The study is a collaboration between the Center for Psychiatry Research (CPF) at the Department of Clinical Neuroscience and the Stockholm Health Services, Region Stockholm, including the Department of Cardiology at Karolinska Universitetssjukhuset, Solna and Psykiatri Sydväst, OCD-programmet at Karolinska Universitetssjukhuset, Huddinge. The screening, initial assessment, and follow-up assessments will take place at an outpatient clinic at the Department of Cardiology, Karolinska Universitssjukhuset, Solna, while the psychoeducation group sessions and exercise sessions will be held at Karolinska Universitetssjukhuset, Huddinge.

## PARTICIPANTS AND SAMPLE SIZE

Participants will be adults meeting criteria for OCD with increased cardiometabolic risk (see definition below; inclusion criterion 3). All inclusion and exclusion criteria are described below. Since the aim of the study is to primarily evaluate the feasibility of the intervention, we do not aim for it to be powered to reveal statistically significant changes in the measures of cardiometabolic risk factors. We aim to include 30 participants to test the feasibility of the intervention. This is a rounded up figure based on the sample sizes in the pilot study by Abrantes et al. (2017) exploring the effect of exercise vs. health education on OCD symptom severity (n=28 in each arm).

## INCLUSION AND EXCLUSION CRITERIA

### Inclusion criteria

1. Aged 18 years or older.

- Confirmed by the participant or assessor at the telephone screening and at the psychiatric assessment.

1. Meeting current criteria for OCD as confirmed by the initial psychiatric evaluation.
   - Screened on the phone and confirmed by the assessor at the initial psychiatric assessment.
2. At least 3 of the following cardiometabolic risk factors:
   1. **Risk alcohol consumption:** >9 units/week or >4 units/occasion for women, >14 units/week or >5 units/occasion for men (Socialstyrelsen, 2018). The information will be collected by asking two self-reported questions: *how many units do you drink every week? how often do you drink 4 (women) or 5 (men) units or more on the same occasion?*
      - Screened on the phone and confirmed by the study nurse at the initial cardiometabolic evaluation.
   2. **Physical inactivity:** <150 min activity/week moderate intensity (i.e., heart beats faster, breathing is faster but able to talk) or <75 min high intensity (i.e., pulse is high, shortened breath, difficulties talking) (World Health Organization, 2020). Evaluated by asking two self-reported questions concerning frequency of weekly moderate or high intensity physical activity and non-exercise physical activity.
      - Screened of the phone and confirmed by the study nurse at the initial cardiometabolic evaluation.
   3. **Unhealthy diet:** Evaluated by asking 5 self-reported questions regarding food habits developed by the Swedish National Board of Health and Welfare, including consumption of vegetables, fruit, fish, sweets/snacks, and breakfast habits (Socialstyrelsen, 2018).
      - Screened on the phone and confirmed by the study nurse at the initial cardiometabolic evaluation.
   4. **Tobacco use (smoking/snuff):**  >1 cigarette/day. Evaluated by asking the following self-reported questions: *do you smoke* (*or did you quit during the last 6 months)? / do you use snuff (or did you quit during the last 6 months)?* If yes: *how many cigarettes per day? / how much snuff?(Socialstyrelsen, 2018)*
      - Screened of the phone and confirmed by the study nurse at the initial cardiometabolic evaluation.
   5. Current or previous **cardiovascular disorder** (except myocardial infarction or stroke within the last 6 months, see exclusion criteria below).

- Screened on the phone and confirmed by the assessor at the initial cardiometabolic evaluation via self-reported interview.
  1. **Abdominal obesity:** Waist circumference **>**88 cm in women, >102 cm in men, according to criteria from the World Health Organization (2011) or sagittal abdominal diameter (SAD): >20 cm in women and >22 cm in men (Risérus et al., 2010).
- Confirmed by the study nurse at the initial cardiometabolic evaluation. Waist circumference measured in a standing position, midway between the lower rib margin and the iliac crest. SAD measured in a supine position at the nearest 0,1 cm with a ruler and a water lever or a calliper, at the level of the umbilical.
  1. **Overweight or obese:** BMI >25 according to criteria from the World Health Organization (World Health Organization, 1995).
- Screened on the phone and confirmed by the study nurse at the initial cardiometabolic evaluation, by calculating the Quetelet index (weight/height^2^). Weight measured on a calibrated scale to the nearest 0.1 kg, height measured barefoot or with thin socks to the nearest 0.1 cm.
  1. **Hypertension:** Systolic blood pressure over 140 mmHg and diastolic blood pressure over 90 mmHg or being currently on medication for hypertension.
- Screened on the phone and confirmed by the study nurse at the initial cardiometabolic evaluation. Systolic and diastolic blood pressure will be measured in a seated position after ten minutes of rest, with a standard sphygmomanometer. The measurement will be taken twice with 1 min between measures, according to the gold standard for measuring blood-pressure (Williams et al., 2018).
  1. **Dyslipidemia:** Elevated total or LDL cholesterol levels or as a high ratio between low density lipoprotein (LDL) and high density lipoprotein (HDL). Total >5.0 mmol/L, LDL >3.0 mmol/L, At risk >1.4 mmol/L (Visseren et al., 2021).
- Confirmed by the study nurse at the initial cardiometabolic evaluation, after analyzing blood sample collected after >8 h fast.
  1. **Impaired glucose tolerance (“prediabetes”):**
     - Confirmed by the study nurse at the initial cardiometabolic evaluation by performing an 2 hour oral glucose tolerance test (OGTT). Reference levels for impaired glucose tolerance is between >7.8 and <11.1 mmol/l according to WHO criteria (World Health Organization, 2006).
  2. **Type 2 diabetes mellitus:** Previous clinical diagnosis or fasting blood glucose levels over 7.0 mmol/l or an OGTT with a 2-hour level 11.1 mmol/l or over.
     - Screened on the phone and confirmed by the study nurse at the initial cardiometabolic evaluation by collecting fasting (>8h) blood sample.

### exclusion criteria

1. Inability to understand and communicate in Swedish.
   - Confirmed at the first phone screening.
2. Inability to travel to Stockholm for the duration of the intervention.
   - Screened on the phone and/or confirmed by the assessor at the initial psychiatric evaluation.
3. Inability to consistently attend the sessions involved in the intervention.
   - Screened on the phone and/or confirmed by the assessor at the initial psychiatric evaluation.
4. Impairing symtoms of OCD that can interfere with participation in the lifestyle intervention (e.g., inability to travel to group and exercise sessions).
   - Screened on the phone and confirmed by assessor at the initial psychiatric evaluation.
5. Intellectual disabilities or a diagnosis of a psychiatric disorder(s) that can interfere with the intervention, such as acute psychotic or bipolar disorders or severe depression.
   - Screened on the phone and confirmed by the assessor at the initial psychiatric evaluation.
6. A clinical diagnosis of an eating disorder or an alcohol or drug use disorder.
   - Screened on the phone and confirmed by the assessor at the initial psychiatric evaluation.
7. Suicidal risk that may interfere with the intervention.
   - Screened on the phone and confirmed by the assessor at the initial psychiatric evaluation (suicide items on the MINI interview).
8. Pregnancy or breastfeeding.
   - Screened on the phone and confirmed by the assessor at the initial psychiatric evaluation or the cardiometabolic evaluation.
9. Myocardial infarction or stroke within the last 6 months.
   - Screened on the phone and confirmed by the assessor at the initial cardiometabolic evaluation.
10. Cardiovascular risk measures siginificantly over the normal range: e.g., severe hypertension (blood pressure above ≥180 mmHg systolic or ≥110 mmHg diastolic) that makes participation in the intervention contraindicated.
    - Confirmed by the assessor at the initial cardiometabolic evaluation.
11. Initiation or adjustment of any cardiometabolic medication (e.g., blood pressure or blood lipids lowering mediation) within 3 months prior to assessments.
    - Screened on the phone and/or confirmed by the assessor at the initial cardiometabolic evaluation.

# Procedure

## Recruitment

### RECRUITMENT SOURCES

Participant recruitment will be broad in order to ensure a good representation of OCD cases and maximize generalizability of the results. Sources will include adult mental health clinics in Stockholm (including the two adult specialist outpatient OCD clinics: Psykiatri Sydväst, OCD-programmet and Psykiatri Nordväst, Ångestenheten), patient organizations such as the *Svenska OCD-förbundet*, and the media, including online social media and advertisements in traditional newspapers and similar outlets.

### SCREENING AND ENROLLING

Potential participants that have expressed interest in the study will be first contacted by telephone by the study coordinator for an initial screening of the inclusion/exclusion criteria. Questions will include: OCD diagnosis and OCD symptom severity; psychiatric history; current psychological treatment; cardiometabolic symptoms, including self-reported weight and height to calculate the BMI, brief medical history (cardiovascular disorders, type 2 diabetes), and current medication; and lifestyle habits, including physical activity, smoking/snuff use, alcohol consumption, and dietary habits, which will be screened through a short questionnaire, based on guidelines from the Swedish National Board of Health and Welfare (Socialstyrelsen, 2018).

Participants that are still considered suitable after the first phone screening, will be invited for a face-to-face at Karolinska Universitetssjukhuset, Solna or phone assessment in which the study coordinator will confirm the diagnosis of OCD via the OCD-RD, a short diagnostic interview originally based on the Structured Clinical Interview (SCID) for DSM-IV, which aligns with the DSM-5 criteria for OCD and related disorders. Additionally, severity of the OCD symptoms will be measured by the Yale-Brown Obsessive-Compulsive Scale (Y-BOCS). Comorbid psychiatric disorders and suicide risk will be assessed by means of the MINI (Mini-International Neuropsychiatric Interview;(Sheehan et al., 1998) in order to check whether other inclusion/exclusion criteria are met. This interview will last around 1-1.5 hours.

For those participants with severe OCD that can interfere with the ability to participate in the lifestyle intervention or that have not received optimal treatment for OCD, appropriate referrals will be arranged.

If, after the psychiatric interview, participants still seem potentially suitable for inclusion, they will be invited to an assessment session at the Department of Cardiology at Karolinska Universitetssjukhuset, Solna where a study nurse specialised in cardiovascular disorders will confirm whether current cardiometabolic risk is present, as per the inclusion criteria above. Data will be collected by means of a semi-structured interview, anthropometric measurements, and a blood test. This assessment will last about 1 hour.

Participants meeting all inclusion/exclusion criteria after the psychiatric and cardiometabolic risk assessments will be informed about the study on the phone and offered formal participation. We will provide written information about the study and get written consent from all participants.

When the consent form has been signed by the participant and received by the study coordinator, and all baseline measures have been completed (see measures and assessment points in section 7), the participant will be assigned a trial ID and enrolled in the study. The signed consent form will be retained in a locked file cabinet. A log will be kept matching screening IDs with trial IDs. Since a full treatment group (6-10 participants) needs to be recruited before the intervention starts, participants will be put on a waiting list after being included and before commencing treatment until a number of participants large enough to start a group has been included. The first individual session with the study coordinator/clinical psychologist (see 6.2 below for details) will be offered up to one week before the start of the groups.

If the participant is not included in the trial (regardless of the reason), but still requires clinical attention, our team will initiate a referral to other suitable services, whenever possible.

The flow of participants, from initial screening to enrolment and inclusion in the intervention, is depicted in **Figure 1** below.

**Figure 1.** Participant flow.

Three-month assessment

(n=x)

Telephone screening

(n=x)

Face-to-face/phone psychiatric evaluation (n=x)

In-person cardiometabolic risk evaluation (n=x)

Included

(n=x)

**Lifestyle intervention**

Post-intervention assessment

(n=x)

Excluded:

-Did not meet preliminary inclusion criteria (n=x)

-Declined participation (n=x)

Excluded:

-Did not meet inclusion criteria (n=x)

-Mention specific reason for exclusion (n=x)

-Declined participation (n=x)

Excluded:

-Did not meet inclusion criteria (n=x)

-Mention specific reason for exclusion (n=x)

-Declined participation (n=x)

## lifestyle intervention

The lifestyle intervention consists of three components: one initial individual session, 6 group educational session, and 12 group exercise sessions.

Intervention begins with one **initial individual session** with the study coordinator (a clinical psychologist) and a nurse specialised in cardiovascular disorders and lifestyle habits to create a personal plan and set up goals for a change of lifestyle habits, based on the baseline evaluation of their clinical characteristics and cardiometabolic risk, which includes the self-reported measures as well as the physiological and laboratory measures.

Further, the participants will then take part in **six manualized group educational sessions** of 1,5-hours each every other week. Sessions will be led/facilitated by the study coordinator (a clinical psychologist with extensive experience working with OCD patients) and a nurse specialised in cardiovascular disorders and lifestyle habits. Each session will consist of a lecture on a certain topic. The sessions will be designed to actively engage participants and encourage group discussions. Between-session homework will be assigned at each occasion. An outline of the content of each group session is summarized in **Table 1**. The group sessions are based on a previous lifestyle intervention evaluated for patients with cardiovascular risk free of psychiatric conditions (Lidin et al., 2018) and adapted for individuals with OCD for the current study. At these sessions, participants will be given a food and activity journal where they will register what they eat, their daily physical activity, and sedentary time so that they can track their changes.

**Table 1**. Content of the intervention group sessions.

| **Session number and main session topic** | **Summarized content** | **Homework/Worksheet** |
| --- | --- | --- |
| 1. OCD education | OCD education (e.g., features, co-occurring conditions, risk factors). How OCD can have a deleterious impact on lifestyle (e.g., obsessions may lead to being housebound and not practicing regular exercise). | - Register OCD symptoms and how they affect lifestyle factors.  - Register daily physical activity and food intake. |
| 1. Overall lifestyle and health | What are unhealthy lifestyles, advice on how to replace them with healthier choices. | - Decide on and try at least one healthier choice: e.g., take stairs instead of elevator, walk/cycle instead of drive, change to low-fat dairy product or a non-sweetened product.  - Register daily physical activity and food intake. |
| 1. Physical activity and sedentary behavior | Defining sedentary behavior and physical activity, health benefits of exercise. Advice to avoid sedentary behavior and increase physical activity. | - Register daily physical activity and food intake. |
| 1. Food habits and alcohol use | Advice on healthy food patterns based on the Nordic Nutrition Recommendations (2012): a dietary pattern rich in vegetables, fruits, fibres, and reduced saturated fats salt and red meat.  Risks of alcohol consumption and advice to quit drinking will be offered. | - Decide on and try at least one healthier food choice: e.g., change to low-fat dairy product or a non-sweetened product, increase vegetable or fruit intake.  - Register daily physical activity, food intake, and alcohol consumption. |
| 1. Smoking, stress, and sleep | Advice on how to stop smoking (if applicable to any group members).  Anti-stress methods (e.g., breathing exercises) and effects on cardiovascular risk will be introduced.  Sleep disorders and negative impact on health will be addressed. Psychoeducation on sleep hygiene. | - For smokers: Follow recommendations from slutarokalinjen.se.  - Practice of breathing and relaxation.  - Changes to improve sleep habits, if necessary.  - Register daily physical activity, food intake, and alcohol consumption. |
| 1. Behavioral change | Practical advice regarding change, motivation, and failure.  Goals setting and relapse prevention. | - Create a plan on how to maintain a healthier lifestyle. |

During the weeks without group educational sessions, the participants will be contacted on the phone for a brief individual follow-up. The participant’s progress towards their personal goals will be assessed, as well as their potential obstacles to reach these goals.

The **group exercise sessions** will be held weekly during the 12 weeks of the intervention; on those weeks with educational sessions, these will be followed by an exercise session. These exercise sessions will be done in the context of the *Braining-project*. Braining consists of group exercise sessions led by mental health staff at Karolinska Universtetssjukhuset, Huddinge. The exercise is of medium to vigorous intensity level and includes both aerobic and resistance training. Each session lasts 30 to 45 minutes. Participation in a minimum of one group exercise session per week is required. However, participants will have the opportunity and will be encouraged to attend more *Braining* sessions (already ongoing at the hospital for other patients with a range of mental disorders) in order to increase the weekly time dedicated to moderately intense physical activity.

After the 12 weeks of lifestyle intervention, participants will be invited to 3 monthly booster sessions (at 1, 2, and 3 months post intervention) to help them maintain their behavioral changes.

# Measures and measurement points

Primary and secondary outcome measures are listed below. **Table 2** describes the assessment points for each one of the measures.

## PRIMARY OUTCOME MEASURES

***Acceptability and feasibility measures*** – collected by the research team or self-reported, as appropriate.

- **Ease of participant recruitment.** This will be assessed by exploring the recruitment rate, number of excluded patients and reasons for exclusion, participant refusals and causes for refusal, and number of patients who declined to participate and reasons why.
- **Adherence to the intervention.** This will be explored through the number of sessions attended.
- **Treatment satisfaction.** It will be measured with The Client Satisfaction Questionnaire (Attkisson, 1994). The CSQ-8 consists of 8 items (quality of service, kind of service, met needs, recommend to a friend, amount of help, deal with problems, overall satisfaction, and come back). Each item is scored on a 4 point Likert scale. Total score range from 8 to 32, where higher scores indicate greater satisfaction. The CSQ-8 has been shown to have high reliability and validity in a mental-health clinical context. The wording of the measure has been slightly modified to match the characteristics of this study.
- **Treatment credibility.** It will be measured with a short questionnaire developed by the research team. It consists of 3 items, scored on a 5-point Likert scale, from 0 to 4: how well the intervention suits people with OCD, how much improvement they expect from the intervention, and how motivated they feel to work with lifestyle change.
- **Attrition rates.** Number of drop-outs will be counted and reasons for disengaging and barriers to participate will be explored via a telephone interview with the study coordinator after dropping out (if the participant dropping out agrees).

## secondary OUTCOME MEASURES

***Lifestyle habits*** – self-reported.

- Risk consumption of alcohol:
  - The Alcohol Use Disorder Identification Test for Consumption, AUDIT-C (Bush et al., 1998): It is an abbreviated version of the AUDIT interview (Johnson et al., 2013). It has demonstrated similar accuracy to detect risk consumption as the full 10 item interview (Kriston et al., 2008).
- Physical activity and sedentary behavior:
  - International Physical Activity Questionnaire (IPAQ), short form (Craig et al., 2003). The IPAQ is one of the most widely used physical acitivity questionnaire, consisting of 7 items concerning physical activity during the last 7 days.
  - Participants will also be handed accelerometers to track physical activity: intensity and duration of activity. The device can also detect time spent sitting and physical inactivity. The participants will be asked to wear the accelerometers for seven consecutive days at different time points (see Table 2).
- Food habits:
  - A short questionnaire developed by the Swedish National Board of Health and Welfare including 5 items covering consumption of vegetables, fruit, fish, sweets/snacks and breakfast habits.
- Smoking:
  - Evaluated by asking the following self-reported questions *do you smoke/ do you use snuff?* If yes: *how many cigarettes per day? / how much snuff?*
- Stress:
  - Perceived Stress Scale (PSS)(Cohen et al., 1983). 10 items to evaluate if the individual has perceived life as uncontrollable, unpredictable and overloading over the last month.
- Sleep pattern:
  - Insomnia Severity Scale (ISI) (Bastien et al., 2001). Consists of 7 items that are rated on a 5 point Likert scale to evaluate sleep problems and severity of symptoms.

***Cardiometabolic risk –*** collected by the study nurse.

Physiological measures including:

1. Waist circumference: This will be measured in a standing position, midway between the lower rib margin and the iliac crest.
2. Sagittal abdominal diameter (SAD): Measured in a supine position at the nearest 0,1 cm with a ruler and a water lever or a calliper, at the level of the umbilical.
3. Weight (in kilograms measured to the nearest 0.1 kg).
4. Body Mass Index (BMI): Height (in meters measured to the nearest 0.1 cm) and weight (in kilograms measured to the nearest 0.1 kg) to calculate the individual’s BMI.
5. Body composition: The measurement of body fat in relation to body mass, by conducting a bio-impedance analysis.
6. Systolic and diastolic blood pressure will be measured in a seated position after ten minutes of rest, with a standard sphygmomanometer. The measurement will be taken twice with 1 min between measures, according to the gold standard for measuring blood-pressure (Williams et al., 2018). The means of the two SBP and DBP measurements will be calculated to be used in the analyses.
7. Resting heart rate will be measured in a seated position following 5-minute rest.

Blood will be drawn after overnight fast (minimum 8 h). Participants who did not fast prior to the blood test will be rescheduled. Fasting blood samples will be analyzed according to local routines at Karolinska Universitetssjukhuset, Solna. The following parameters will be analyzed:

1. Total cholesterol (mmol/l), S-low density lipoprotein cholesterol (mmol/l), and S-high density lipoprotein cholesterol (mmol/l).
2. Fasting triglycerides.
3. P-glucose (mmol/l).
4. HbA1c (%). *Glycated haemoglobin:* This reflects average plasma glucose over the previous eight to 12 weeks, and does not require fasting. Can be used in the diagnosis of diabetes.
5. Oral glucose tolerance test (OGTT).
6. Inflammatory and genetic biomarkers (e.g high-sensitive CRP, white blood cell telomere length).
7. Framingham risk score: This is a gender-specific algorithm used to estimate the 10-year cardiovascular risk of an individual, and can be used for individuals with previous and non-previous CVD. It is based on age, smoking, systolic BP and medical treatment for hypertension or not, total cholesterol, HDL-cholesterol, and occurrence of diabetes type 2. The 10-year risk for women can be calculated as 1-0.95012^exp(ΣßX – 26.1931)^ where ß is the regression coefficient and X is the level for each risk factor; the risk for men is given as 1-0.88936^exp(ΣßX – 23.9802)^ (D'Agostino et al., 2008).

***Other:***

- OCD-specific measures:
  - Clinician-reported: Y-BOCS (Goodman et al., 1989). The gold standard measure to assess OCD symptom severity.
  - Self-reported: Obsessive-Compulsive Inventory – 12 (Abramovitch et al., 2021). 12 items on OCD symptoms rated on a 5 point Likert scale.
- Depressive symptoms:
  - Patient Health Questionnaire, PHQ-9 (Kroenke et al., 2001). Self-reported, 9-items scale to assess symptoms of depression.
- Functional impairment:
  - WSAS: Work and Social Adjustment Scale (Mundt et al., 2002). A reliable measure of impairment in functioning, in terms of work, home management, leisure activities, and relationships.
- Health-related quality of life:
  - EQ-5D (Rabin & de Charro, 2001). The participant rates their own health status in five dimensions: mobility; self-care; usual activities; pain/discomfort; anxiety/depression, within three levels of severity: no problems; moderate problems; severe problems. The scale also comprises a rating of their overall health status on a scale from 0-100 (0 = worst imaginable health; 100 = best imaginable health), the EQ-VAS scale.

## adverse events

Adverse events will be closely monitored. Participants will be asked to fill out a short questionnaire designed for this study regarding potential adverse events (e.g., increased anxiety, headaches, increased tiredness, musculoskeletal pain). They will also be able to write own examples on negative experiences connected to participation in the intervention. The questionnaire will be administered at mid- and post-intervention. Adverse events will also be monitored by group leaders at all educational group sessions and checked at the brief telephone follow-up the weeks without educational group sessions.

## Participants’ experiences

Participants’ experiences of taking part in the lifestyle intervention will be explored by a more thorough qualitative interview on the phone/video call or face-to-face according to participant’s choice. The interview will consist of two parts. The first part will explore the experience of participating in the trial to be able to further improve the lifestyle intervention.The second part focus on how OCD symptoms affect lifestyle habits and the ability to make lifestyle changes, as well as experiences of seeking and receiving somatic health care (see list of questions in Appendix A at the end of this document) compiled by the research group. All participants will be asked for participation in this qualitative part, but completion will be optional and independent from participation in the described intervention.

**Table 2.** Primary and secondary outcome measures and assessment time-points.

| Measure | | Baseline | Mid-intervention | Post-intervention | 3 months |  |
| --- | --- | --- | --- | --- | --- | --- |
| ***Feasibility measures:*** | |  |  |  |  |  |
| CSQ-8 | |  |  | x |  |  |
| Credibility questionnaire | | x |  |  |  |  |
| Adverse events questionnaire | |  | x | x |  |  |
| ***Physiological measures:*** | |  |  |  |  |  |
| Waist circumference | | x |  | x | x |  |
| Sagittal abdominal diameter (SAD) | x |  | x | x |  |  |
| Weight | | x |  | x | x |  |
| Body mass index (BMI) | | x |  | x | x |  |
| Bio-impedance | | x |  | x | x |  |
| Blood pressure (systolic /diastolic) | | x |  | x | x |  |
| Resting heart rate | | x |  | x | x |  |
| Framingham risk score | | x |  | x | x |  |
| ***Laboratory measures:*** | |  |  |  |  |  |
| Fasting cholesterol | | x |  | x | x |  |
| Fasting triglycerides | | x |  | x | x |  |
| Fasting plasma glucose | | x |  | x | x |  |
| OGTT | | x |  |  | x |  |
| Inflammatory and genetic biomarkers | | x |  | x | x |  |
| ***Lifestyle habits:*** | |  |  |  |  |  |
| Alcohol, AUDIT-C, 3 items | | x |  | x | x |  |
| Physical acitivity, IPAQ, 7 items | | x |  | x | x |  |
| Daily activity, accelerometer | | x |  | x | x |  |
| Eating habits, 5 items | | x |  | x | x |  |
| Smoking/Snuff, 2 items | | x |  | x | x |  |
| Stress, PSS-10, 10 items | | x |  | x | x |  |
| Sleep, ISI, 7 items | | x |  | x | x |  |
| ***OCD and psychiatric measures:*** | |  |  |  |  |  |
| Y-BOCS | | x |  | x | x |  |
| OCI-12 | | x |  | x | x |  |
| WSAS | | x |  | x | x |  |
| EQ-5D | | x |  | x | x |  |
| PHQ-9 | | x |  | x | x |  |

Abbreviations: AUDIT-C=Alcohol Use Disorders Identification Test, CRP= C-Reactive Protein, CSQ-8=Client Satisfaction Questionnaire, EQ-5D= The EuroQol five-dimensional questionnaire, ISI= Insomnia Severity Scale, IPAQ=International Physical Activity Questionnaire, OCI- 12= Obsessive Compulsive Inventory, OGTT= Oral glucose tolerance test, PHQ-9= Patient Health Questionnaire, PSS-10= Perceived Stress Scale, Y-BOCS= Yale-Brown Obsessive-Compulsive Scale, WSAS=Work and Social Adjustment Scale.

# Data management

Data will be collected manually in Case Report Forms (CRFs). The CRFs will not include the participant’s name, instead, the trial identification number will be used for identification. The CRFs will be stored securely in a locked file cabinet. The delegation log will identify all staff with responsibilities for data collection and handling, including those who have access to the trial database.

# STATISTICAL ANALYSIS

Summary of variables (e.g., frequencies, percentages) and descriptive statistics (e.g., means, standard deviations) will be presented as appropriate. To detect significant within-group pre to post intervention and pre to 3-month follow-up changes, we will perform linear mixed-effects regression analyses to take into account individual differences in baseline status and response to the intervention. The results will be stratified by gender, power allowing.

# Public and patient involvement

All phases of the project will take into account patients’ perspectives, to ensure that the intervention is feasible, safe, and acceptable for the patients. During the development of the lifestyle intervention, focus groups will be conducted where patients will be represented by members of the Swedish OCD foundation (*Svenska OCD-förbundet*). Patient representatives will be able to provide feedback on the eligibility criteria and the intervention, among other study elements, and suggest modifications to suit the needs of the individuals with OCD. To our knowledge, this is the first time a lifestyle intervention targeting cardiometabolic risk factors is developed and tested for OCD and feedback from patients will be of great importance throughout the project.

Participants enrolled in the study will continuously have the opportunity to provide feedback and share their views and experiences on how the intervention and procedures can be improved. Participants will also be able to give more specific feedback of the intervention and give suggestions for improvement in an open question. They will also be asked if they agree to be contacted via phone for a more thorough qualitative interview regarding their experience of taking part in the lifestyle intervention (e.g., what elements have been more/less helpful, what aspects would they introduce/change/remove to improve the intervention).

We plan to disseminate the results of the pilot study to study participants, the *Svenska* *OCD-förbundet*, and the general public.

# Data sharing

We may potentially share de-identified data from the study with other OCD researchers around the world. The use would primarily be to combine outcome data for potential meta-analyses in this research field. The possibility of future data sharing is mentioned in the informed consent form.

# Ethical considerations

All participants will receive a thorough psychiatric and cardiometabolic risk assessments before enrolment in the study. Those not included in the study will be referred to or recommended other treatment options. Participants will be informed that they can withdraw their participation from the study at any time.

Even though the lifestyle intervention tested in this trial has never been evaluated for an OCD population, it is based on guidelines from the Swedish National Board of Health and Welfare on how to target unhealthy lifestyle habits and previous interventions for other mental health populations and the general population. Therefore, the proposed intervention is deemed safe from the clinical point of view.

Adverse events will be monitored throughout the trial, but risk for participants is considered to be low. A previous study on a lifestyle intervention for severe mental illness showed fewer medical hospitalizations in intervention group (n=104), and no serious study-related adverse events occurred (Green et al., 2015). On the other hand, a larger trial (n=810) of a lifestyle intervention targeting hypertension in the general population (PREMIER trial) reported 32 musculoskeletal injuries and one myocardial infarction in the two intervention groups (Appel et al., 2003). These potential side effects will be carefully monitored. Other short-term adverse events that can be experienced by some participants are increased OCD symptoms and anxiety. Participants will learn more on how to manage these symptoms during group sessions and group leaders are experienced in working with individuals with these disorders, which will contribute to containing these symptoms during the duration of the intervention.

## The Covid-19 pandemic

This trial will follow the recommendations of the Swedish public health authority (*Folkhälsomyndigheten*) and adopt/modify procedures according to these, if relevant/necessary.

# Implications

Cardiometabolic diseases are responsible for one third of all global deaths (World Health Organization, 2017). Additionally, as described above, our previous research has established that patients with OCD have an increased risk for these disorders, which makes them a particularly vulnerable group important to target. Unique environmental factors such as unhealthy lifestyle habits are likely to play a role in the development of these outcomes. In fact, behavioral and metabolic risks have shown to drive most deaths and disability in Sweden in the Global Burden of Disease study of 2019, with tobacco, dietary risks, and alcohol use as most important behavioral factors, and high fasting plasma glucose, high blood pressure, high body mass index, high LDL, and impaired kidney function as most important cardiometabolic factors.

Scientific evaluations of lifestyle interventions in clinical practice are still limited and, although some attempts at assessing these interventions have been carried out in other mental disorders (Cabassa et al., 2010), to the best of our knowledge, this will be the world’s first study to specifically target lifestyle habits in individuals with OCD. We will do so in close collaboration with the Swedish OCD patient organization (*Svenska OCD-förbundet*), to ensure that we are developing an intervention that the patients themselves will want to use.

If the intervention proves to be feasible, safe, and accepted by participants, we aim to conduct a fully powered randomized controlled trial to establish the efficacy and cost-effectiveness of this intervention. Eventually, if the intervention is shown to be effective in reducing cardiometabolic risk and does so in a cost-effective manner, we will aim to implement it in routine clinical care upon termination of the project.

# Finance

This trial is financed by the Swedish Research Council for Health, Working Life and Welfare, FORTE (grant number 2019-00438), the Swedish Research Council (grant number 2022-005109), Region Stockholm / ALF Medicin (grant number 20200139), Hjärt-Lungfonden (grant numbers 20210493 and 20220899), and the Committee for Research at Karolinska Institutet (grant numbers 2020-01361 and 2022-01675).

# Publications

We will communicate our results to the scientific community by publishing the results in high impact scientific journals. In order to maximize visibility of our results, we will pursue open access formats. Additionally, results will be presented at a number of national and international key conferences delivering results directly to active members of the scientific community. (e.g., American Psychiatric Association) and other professional venues.

# References

Abramovitch, A., Abramowitz, J. S., & McKay, D. (2021, 2021/04/01/). The OCI-12: A syndromally valid modification of the obsessive-compulsive inventory-revised. *Psychiatry Research, 298*, 113808. https://doi.org/https://doi.org/10.1016/j.psychres.2021.113808

Abrantes, A. M., Brown, R. A., Strong, D. R., McLaughlin, N., Garnaat, S. L., Mancebo, M., Riebe, D., Desaulniers, J., Yip, A. G., Rasmussen, S., & Greenberg, B. D. (2017, 2017/11/01/). A pilot randomized controlled trial of aerobic exercise as an adjunct to OCD treatment. *General Hospital Psychiatry, 49*, 51-55. https://doi.org/https://doi.org/10.1016/j.genhosppsych.2017.06.010

American Psychiatric Association. (2007). *Practice guideline for the treatment of patients with OCD.* . APA.

American Psychiatric Association. (2013). *Diagnostic and Statistical Manual of Mental Disorders. Fifth edition.* (5th ed.). American Psychiatric Association. .

Appel, L. J., Champagne, C. M., Harsha, D. W., Cooper, L. S., Obarzanek, E., Elmer, P. J., Stevens, V. J., Vollmer, W. M., Lin, P. H., Svetkey, L. P., Stedman, S. W., & Young, D. R. (2003, Apr 23-30). Effects of comprehensive lifestyle modification on blood pressure control: main results of the PREMIER clinical trial. *JAMA, 289*(16), 2083-2093. https://doi.org/10.1001/jama.289.16.2083

Attkisson, C. C., & Greenfield, T. K. (1994). Client Satisfaction Questionnaire-8 and Service Satisfaction Scale-30. In M. E. Maruish (Ed.), *The use of psychological testing for treatment planning and outcome assessment* (pp. 402–420). Lawrence Erlbaum Associates, Inc.

Bastien, C. H., Vallières, A., & Morin, C. M. (2001, Jul). Validation of the Insomnia Severity Index as an outcome measure for insomnia research. *Sleep Medicine, 2*(4), 297-307. https://doi.org/10.1016/s1389-9457(00)00065-4

Brown, R. A., Abrantes, A. M., Strong, D. R., Mancebo, M. C., Menard, J., Rasmussen, S. A., & Greenberg, B. D. (2007, Jun). A pilot study of moderate-intensity aerobic exercise for obsessive compulsive disorder. *J Nerv Ment Dis, 195*(6), 514-520. https://doi.org/10.1097/01.nmd.0000253730.31610.6c

Bush, K., Kivlahan, D. R., McDonell, M. B., Fihn, S. D., & Bradley, K. A. (1998, Sep 14). The AUDIT alcohol consumption questions (AUDIT-C): an effective brief screening test for problem drinking. Ambulatory Care Quality Improvement Project (ACQUIP). Alcohol Use Disorders Identification Test. *Arch Intern Med, 158*(16), 1789-1795. https://doi.org/10.1001/archinte.158.16.1789

Cabassa, L. J., Ezell, J. M., & Lewis-Fernandez, R. (2010). Lifestyle Interventions for Adults With Serious Mental Illness: A Systematic Literature Review. *Psychiatric Services, 61*(8), 774-782. https://doi.org/10.1176/appi.ps.61.8.774

Cohen, S., Kamarck, T., & Mermelstein, R. (1983, Dec). A global measure of perceived stress. *Journal of Health and Social Behavior, 24*(4), 385-396.

Craig, C. L., Marshall, A. L., Sjöström, M., Bauman, A. E., Booth, M. L., Ainsworth, B. E., Pratt, M., Ekelund, U., Yngve, A., Sallis, J. F., & Oja, P. (2003, Aug). International physical activity questionnaire: 12-country reliability and validity. *Medicine and Science in Sports and Exercise, 35*(8), 1381-1395. https://doi.org/10.1249/01.Mss.0000078924.61453.Fb

D'Agostino, R. B., Sr., Vasan, R. S., Pencina, M. J., Wolf, P. A., Cobain, M., Massaro, J. M., & Kannel, W. B. (2008, Feb 12). General cardiovascular risk profile for use in primary care: the Framingham Heart Study. *Circulation, 117*(6), 743-753. https://doi.org/10.1161/circulationaha.107.699579

Fernandez de la Cruz, L., Rydell, M., Runeson, B., D'Onofrio, B. M., Brander, G., Ruck, C., Lichtenstein, P., Larsson, H., & Mataix-Cols, D. (2017, Nov). Suicide in obsessive-compulsive disorder: a population-based study of 36 788 Swedish patients. *Mol Psychiatry, 22*(11), 1626-1632. https://doi.org/10.1038/mp.2016.115

Global burden of 87 risk factors in 204 countries and territories, 1990-2019: a systematic analysis for the Global Burden of Disease Study 2019. (2020, Oct 17). *Lancet, 396*(10258), 1223-1249. https://doi.org/10.1016/s0140-6736(20)30752-2

Goodman, W. K., Price, L. H., Rasmussen, S. A., Mazure, C., Fleischmann, R. L., Hill, C. L., Heninger, G. R., & Charney, D. S. (1989, Nov). The Yale-Brown Obsessive Compulsive Scale. I. Development, use, and reliability. *Archives of General Psychiatry, 46*(11), 1006-1011. https://doi.org/10.1001/archpsyc.1989.01810110048007

Green, C. A., Yarborough, B. J. H., Leo, M. C., Yarborough, M. T., Stumbo, S. P., Janoff, S. L., Perrin, N. A., Nichols, G. A., & Stevens, V. J. (2015). The STRIDE Weight Loss and Lifestyle Intervention for Individuals Taking Antipsychotic Medications: A Randomized Trial. *American Journal of Psychiatry, 172*(1), 71-81. https://doi.org/10.1176/appi.ajp.2014.14020173

Isomura, K., Brander, G., Chang, Z., Kuja-Halkola, R., Rück, C., Hellner, C., Lichtenstein, P., Larsson, H., Mataix-Cols, D., & Fernández de la Cruz, L. (2018). Metabolic and Cardiovascular Complications in Obsessive-Compulsive Disorder: A Total Population, Sibling Comparison Study With Long-Term Follow-up. *Biological Psychiatry, 84*(5), 324-331. https://doi.org/10.1016/j.biopsych.2017.12.003

Isomura, K., Sidorchuk, A., Brander, G., Jernberg, T., Rück, A., Song, H., Valdimarsdóttir, U. A., Lichtenstein, P., Larsson, H., Rück, C., Mataix-Cols, D., & Fernández de la Cruz, L. (2021, 2021/03/01/). Risk of specific cardiovascular diseases in obsessive-compulsive disorder. *Journal of Psychiatric Research, 135*, 189-196. https://doi.org/https://doi.org/10.1016/j.jpsychires.2020.12.066

Johnson, J. A., Lee, A., Vinson, D., & Seale, J. P. (2013, Jan). Use of AUDIT-based measures to identify unhealthy alcohol use and alcohol dependence in primary care: a validation study. *Alcoholism, Clinical and Experimental Research, 37 Suppl 1*, E253-259. https://doi.org/10.1111/j.1530-0277.2012.01898.x

Kriston, L., Hölzel, L., Weiser, A. K., Berner, M. M., & Härter, M. (2008, Dec 16). Meta-analysis: are 3 questions enough to detect unhealthy alcohol use? *Annals of Internal Medicine, 149*(12), 879-888. https://doi.org/10.7326/0003-4819-149-12-200812160-00007

Kroenke, K., Spitzer, R. L., & Williams, J. B. (2001, Sep). The PHQ-9: validity of a brief depression severity measure. *Journal of General Internal Medicine, 16*(9), 606-613. https://doi.org/10.1046/j.1525-1497.2001.016009606.x

Lidin, M., Hellenius, M. L., Rydell-Karlsson, M., & Ekblom-Bak, E. (2018, Apr 2). Long-term effects on cardiovascular risk of a structured multidisciplinary lifestyle program in clinical practice. *BMC Cardiovasc Disord, 18*(1), 59. https://doi.org/10.1186/s12872-018-0792-6

Matthews, D. R., Hosker, J. P., Rudenski, A. S., Naylor, B. A., Treacher, D. F., & Turner, R. C. (1985, Jul). Homeostasis model assessment: insulin resistance and beta-cell function from fasting plasma glucose and insulin concentrations in man. *Diabetologia, 28*(7), 412-419. https://doi.org/10.1007/bf00280883

Meier, S. M., Mattheisen, M., Mors, O., Schendel, D. E., Mortensen, P. B., & Plessen, K. J. (2016, Mar). Mortality Among Persons With Obsessive-Compulsive Disorder in Denmark. *JAMA Psychiatry, 73*(3), 268-274. https://doi.org/10.1001/jamapsychiatry.2015.3105

Ministers, N. C. o. (2014). *Nordic Nutrition Recommendations 2012: Integrating Nutrition and Physical Activity*. Nordic Council of Ministers. https://books.google.se/books?id=6lAFrgEACAAJ

Mundt, J. C., Marks, I. M., Shear, M. K., & Greist, J. H. (2002, May). The Work and Social Adjustment Scale: a simple measure of impairment in functioning. *Br J Psychiatry, 180*, 461-464. https://doi.org/10.1192/bjp.180.5.461

NICE. (2006). Obsessive-compulsive disorder *British Psychological Society & Royal College of Psychiatrists*.

Rabin, R., & de Charro, F. (2001, Jul). EQ-5D: a measure of health status from the EuroQol Group. *Ann Med, 33*(5), 337-343. https://doi.org/10.3109/07853890109002087

Rector, N. A., Richter, M. A., Lerman, B., & Regev, R. (2015, 2015/07/04). A Pilot Test of the Additive Benefits of Physical Exercise to CBT for OCD. *Cognitive Behaviour Therapy, 44*(4), 328-340. https://doi.org/10.1080/16506073.2015.1016448

Risérus, U., de Faire, U., Berglund, L., & Hellénius, M. L. (2010). Sagittal abdominal diameter as a screening tool in clinical research: cutoffs for cardiometabolic risk. *Journal of Obesity, 2010*, 757939. https://doi.org/10.1155/2010/757939

Ruscio, A. M., Stein, D. J., Chiu, W. T., & Kessler, R. C. (2008). The epidemiology of obsessive-compulsive disorder in the National Comorbidity Survey Replication. *Molecular psychiatry, 15*(1), 53-63. https://doi.org/10.1038/mp.2008.94

Sheehan, D. V., Lecrubier, Y., Sheehan, K. H., Amorim, P., Janavs, J., Weiller, E., Hergueta, T., Baker, R., & Dunbar, G. C. (1998). The Mini-International Neuropsychiatric Interview (M.I.N.I.): the development and validation of a structured diagnostic psychiatric interview for DSM-IV and ICD-10. *Journal of Clinical Psychiatry, 59 Suppl 20*, 22-33;quiz 34-57.

Socialstyrelsen. (2018). Prevention och behandling vid ohälsosamma levnadsvanor -stöd för styrning och ledning. *In: Socialstyrelsen, editor*. https://www.socialstyrelsen.se/globalassets/sharepoint-dokument/artikelkatalog/nationella-riktlinjer/2018-6-24.pdf

Svetkey, L. P., Harsha, D. W., Vollmer, W. M., Stevens, V. J., Obarzanek, E., Elmer, P. J., Lin, P.-H., Champagne, C., Simons-Morton, D. G., Aickin, M., Proschan, M. A., & Appel, L. J. (2003, 2003/07/01/). Premier: a clinical trial of comprehensive lifestyle modification for blood pressure control: rationale, design and baseline characteristics. *Annals of Epidemiology, 13*(6), 462-471. https://doi.org/https://doi.org/10.1016/S1047-2797(03)00006-1

Visseren, F. L. J., Mach, F., Smulders, Y. M., Carballo, D., Koskinas, K. C., Bäck, M., Benetos, A., Biffi, A., Boavida, J.-M., Capodanno, D., Cosyns, B., Crawford, C., Davos, C. H., Desormais, I., Di Angelantonio, E., Franco, O. H., Halvorsen, S., Hobbs, F. D. R., Hollander, M., Jankowska, E. A., Michal, M., Sacco, S., Sattar, N., Tokgozoglu, L., Tonstad, S., Tsioufis, K. P., van Dis, I., van Gelder, I. C., Wanner, C., Williams, B., & Group, E. S. C. S. D. (2021). 2021 ESC Guidelines on cardiovascular disease prevention in clinical practice: Developed by the Task Force for cardiovascular disease prevention in clinical practice with representatives of the European Society of Cardiology and 12 medical societies With the special contribution of the European Association of Preventive Cardiology (EAPC). *European Heart Journal, 42*(34), 3227-3337. https://doi.org/10.1093/eurheartj/ehab484

Williams, B., Mancia, G., Spiering, W., Agabiti Rosei, E., Azizi, M., Burnier, M., Clement, D. L., Coca, A., de Simone, G., Dominiczak, A., Kahan, T., Mahfoud, F., Redon, J., Ruilope, L., Zanchetti, A., Kerins, M., Kjeldsen, S. E., Kreutz, R., Laurent, S., Lip, G. Y. H., McManus, R., Narkiewicz, K., Ruschitzka, F., Schmieder, R. E., Shlyakhto, E., Tsioufis, C., Aboyans, V., & Desormais, I. (2018, Oct). 2018 ESC/ESH Guidelines for the management of arterial hypertension: The Task Force for the management of arterial hypertension of the European Society of Cardiology and the European Society of Hypertension: The Task Force for the management of arterial hypertension of the European Society of Cardiology and the European Society of Hypertension. *J Hypertens, 36*(10), 1953-2041. https://doi.org/10.1097/hjh.0000000000001940

World Health Organization. (1995). *Physical status: the use of and interpretation of anthropometry, report of a WHO expert committee*.

World Health Organization. (2006). *Definition and diagnosis of diabetes mellitus and intermediate hyperglycaemia: Report of a WHO/IDF consultation.*

World Health Organization. (2011). *Waist circumference and waist-hip ratio: report of a WHO expert consultation*. ISBN: 9789241501491

World Health Organization. (2017). *Cardiovascular diseases (CVDs)*.

World Health Organization. (2020). *WHO guidelines on physical activity and sedentary behaviour*. World Health Organization. https://apps.who.int/iris/handle/10665/336656

Appendix A.

**Intervjuguide- upplevelse av deltagande i ”En strukturerad livsstilsintervention för att minska riskfaktorer för kardiometabola sjukdomar hos personer med tvångssyndrom (OCD)”**

1. Vad tyckte du om omfattningen på interventionen? (12 veckor, gruppträff varannan vecka, gruppträning varje vecka)
2. Hur upplevde du innehållet i gruppträffarna om levnadsvanor?
3. Hur var längden på gruppträffarna?
4. Hur var det att jobba med hemuppgifter?
5. Hur var det att delta i gruppträning?
6. Hur var längden och omfattningen på gruppträningspassen? (30-45 min, 12 veckor)
7. Var passen på lagom nivå?
8. Hur upplevde du att delta i en grupp med andra personer med tvångssyndrom?
9. Vilka delar i interventionen har varit mest hjälpsamt?
10. Vilka delar har varit minst hjälpsamma?
11. Finns det något du skulle vilja förändra/ta bort i interventionen?
12. Finns det något du skulle vilja lägga till för att förbättra interventionen?
13. Har du gjort några förändringar i din livsstil? I så fall, berätta lite om hur processen har varit?
14. Är det något du vill tillägga?

**Intervjuguide - del 2**

1. **Vad fick dig att delta i LIFT-interventionen?**

- Vad hoppades du på att få ut av interventionen?

1. **Upplever du att tvånget har påverkat dina levnadsvanor? I så fall, på vilket sätt?**

- Fysisk aktivitet
- Mat
- Alkohol
- Sömn
- Rökning/snusning

1. **Upplever du att tvånget hindrat dig från att göra förändringar i hälsobeteenden som du velat göra?**

- På vilket sätt?
- Under interventionen eller generellt i livet?

1. **Upplever du att annan psykisk ohälsa eller diagnoser hindrat dig från att göra förändringar i hälsobeteenden som du velat göra?**

- Under interventionen eller generellt i livet?

1. **Har tvånget påverkat din möjlighet att delta aktivt i interventionen?**

- Hade du behövt någon mer anpassning utifrån ditt tvångssyndrom?
- Finns det något annat som har påverkat?

1. **Har du upplevt att tvånget eller din psykiska hälsa överlag har hindrat dig från att få tillgång till kroppslig hälso- och sjukvård?**

- På vilket sätt?
