# Supplementary material for: A structured lifestyle intervention to reduce cardiometabolic risk factors in individuals with obsessive–compulsive disorder: feasibility trial
Source: BJPsych Open. 2025 Aug 1;11(5):e171. doi: 10.1192/bjo.2025.10774 (PMC12344427; doi:10.1192/bjo.2025.10774)
Supplement: Holmberg et al. supplementary material 2 — Holmberg et al. supplementary material [file S2056472425107746sup002.docx]

**Supplementary Methods**

**Extended description of the self-reported preliminary efficacy measures**

***Lifestyle habits***

*Level of physical activity* and *sedentary behavior* were measured with the self-reported International Physical Activity Questionnaire (IPAQ), short form (1), a widely used physical activity questionnaire consisting of seven items concerning physical activity and time spent sitting during the last week. The IPAQ has shown acceptable measurement properties, at least as good as other established self-reports (2). The questionnaire is validated in the Swedish population, showing acceptable criterion validity. (3).

*Dietary habits* were measured by a 5-item questionnaire developed by the Swedish National Board of Health and Welfare covering consumption of vegetables, fruit, fish, sweets/snacks, and breakfast habits (4). The four first questions are summarized into a dietary index score, ranging from 0 to 12 points, where a score ≤4 is considered the cut-off for unhealthy dietary habits (4).

*Alcohol consumption* was measured with the self-reported Alcohol Use Disorder Identification Test for Consumption (AUDIT-C) (5), ranging from 0 to 12 points. A cut-off ≥4 points is indicative of risk consumption of alcohol (6, 7). The AUDIT-C has demonstrated similar accuracy to detect risk consumption as the full 10 item AUDIT interview (8).

*Tobacco use* was evaluated by asking the participants whether they had ever smoked cigarettes or used snuff (smokeless tobacco), if they smoked or used snuff daily, if they smoked or used snuff but not daily, and if they had quit smoking or using snuff. If applicable, participants were also asked to specify the amount of cigarettes/snuff used. For the analysis, smokers and snuff users were merged (tobacco user yes/no).

*Stress* was measured with the 10-item Perceived Stress Scale (PSS) (9). The Swedish version has good psychometric properties (10). The total score ranges from 0-40. The mean score in the Swedish general population is 13.5 points (10).

*Sleep* was measured with the self-reported Insomnia Severity Scale (ISI) (11), consisting of seven items rated on a 5-point Likert scale evaluating sleep problems and their severity. The total score ranges from 0 to 28 points. A cut-off of 11 in clinical samples has been suggested (12). The scale has good psychometric properties (11, 12).

***Mental health and quality of life***

*OCD symptom severity* was measured with the Yale-Brown Obsessive-Compulsive Scale (Y-BOCS) (13) and the Obsessive-Compulsive Inventory – 12 (OCI-12) (14). The Y-BOCS is a clinician-rated semi-structured interview which is the gold standard measure to assess OCD symptom severity. The Y-BOCS consists of 10 items rated from 0 to 4, with a total score from 0 to 40, where a higher score corresponds to more severe OCD. The Y-BOCS has good psychometric properties (13). The OCI-12 consists of 12 self-reported items on OCD symptoms rated on a 5-point Likert scale. The scale has shown good to excellent psychometric properties and good sensitivity and specificity for clinical cut-off scores. Scores between 0 and 12 indicate mild, between 13 and 21 indicate moderate, and between 22 and 48 indicate severe OCD symptoms (14).

*Depressive symptoms* were measured with the Patient Health Questionnaire (PHQ-9) (15). The PHQ-9 is a self-reported, 9-item scale with a total score ranging from 0 to 27 points, with a higher score corresponding to more severe symptoms of depression. In a psychiatric sample, a cut-off ≥13 points has shown good sensitivity and specificity to detect depression (16).

*Functional impairment* was measured with the Work and Social Adjustment Scale (WSAS) (17). The WSAS has five items and measures impairment in different life domains, including work, home management, social leisure activities, private leisure activities, and relationships. Each item is rated on a scale from 0 to 8, total score ranges from 0 to 40. A higher score corresponds to more impairment in functioning. The WSAS has shown excellent psychometric properties (17).

*Quality of life* and *health status* were measured with the EQ-5D-3L (18). The participant rates their own health status in five dimensions (namely mobility, self-care, usual activities, pain/discomfort, and anxiety/depression), within three levels of severity (no problems, moderate problems, and severe problems). Responses to these five dimensions result in a health index score. The scale also comprises a rating of their overall health status on a visual analogue scale from 0-100 (0 = worst imaginable health; 100 = best imaginable health), the EQ-VAS scale. The EQ-5D-3L has good psychometric properties (19).

**Interview guide: Part 1**

1. What did you think about the duration and structure of the intervention? (i.e., 12 weeks, educational group sessions every other week, exercise group sessions every week)
2. What are your thoughts on the content of the lectures on lifestyle habits within the educational group sessions?
3. What did you think about the length of the lectures?
4. How did you find the homework assignments?
5. How did you find to participate in the exercise group sessions?
6. What did you think about the length and content of the exercise group sessions? (i.e., 30-45 minutes/each for 12 weeks)?
7. Were the exercise group sessions at an appropriate level?
8. How was it to participate in a group with other people with obsessive-compulsive disorder?
9. Which parts of the intervention did you find most helpful?
10. Which parts of the intervention did you find least helpful?
11. Is there anything you would like to change or remove from the intervention?
12. Is there anything you would like to add to improve the intervention?
13. Have you made any lifestyle changes? If so, please tell us about how this process has been for you.
14. Is there anything else you would like to add?

**Additional references**

1. Craig CL, Marshall AL, Sjöström M, Bauman AE, Booth ML, Ainsworth BE*, et al.* International physical activity questionnaire: 12-country reliability and validity. Medicine and Science in Sports and Exercise. 2003;35(8):1381-95.

2. Lee PH, Macfarlane DJ, Lam TH, Stewart SM. Validity of the international physical activity questionnaire short form (IPAQ-SF): A systematic review. International Journal of Behavioral Nutrition and Physical Activity. 2011;8(1):115.

3. Ekelund U, Sepp H, Brage S, Becker W, Jakes R, Hennings M*, et al.* Criterion-related validity of the last 7-day, short form of the International Physical Activity Questionnaire in Swedish adults. Public Health Nutrition. 2006;9(2):258-65.

4. Socialstyrelsen. Prevention och behandling vid ohälsosamma levnadsvanor -stöd för styrning och ledning [National Board of Health and Welfare- Prevention and treatment of unhealthy lifestyle habits]. In: Socialstyrelsen, editor. 2018.

5. Bush K, Kivlahan DR, McDonell MB, Fihn SD, Bradley KA. The AUDIT alcohol consumption questions (AUDIT-C): an effective brief screening test for problem drinking. Ambulatory Care Quality Improvement Project (ACQUIP). Alcohol Use Disorders Identification Test. Arch Intern Med. 1998;158(16):1789-95.

6. Lundin A, Hallgren M, Balliu N, Forsell Y. The use of alcohol use disorders identification test (AUDIT) in detecting alcohol use disorder and risk drinking in the general population: validation of AUDIT using schedules for clinical assessment in neuropsychiatry. Alcoholism, Clinical and Experimental Research. 2015;39(1):158-65.

7. Reinert DF, Allen JP. The Alcohol Use Disorders Identification Test: An Update of Research Findings. Alcoholism: Clinical and Experimental Research. 2007;31(2):185-99.

8. Kriston L, Hölzel L, Weiser AK, Berner MM, Härter M. Meta-analysis: are 3 questions enough to detect unhealthy alcohol use? Annals of Internal Medicine. 2008;149(12):879-88.

9. Cohen S. Perceived stress in a probability sample of the United States. The social psychology of health. The Claremont Symposium on Applied Social Psychology. Thousand Oaks, CA, US: Sage Publications, Inc; 1988. p. 31-67.

10. Nordin M, Nordin S. Psychometric evaluation and normative data of the Swedish version of the 10-item perceived stress scale. Scandinavian Journal of Psychology. 2013;54(6):502-7.

11. Bastien CH, Vallières A, Morin CM. Validation of the Insomnia Severity Index as an outcome measure for insomnia research. Sleep Medicine. 2001;2(4):297-307.

12. Morin CM, Belleville G, Bélanger L, Ivers H. The Insomnia Severity Index: Psychometric Indicators to Detect Insomnia Cases and Evaluate Treatment Response. Sleep. 2011;34(5):601-8.

13. Goodman WK, Price LH, Rasmussen SA, Mazure C, Fleischmann RL, Hill CL*, et al.* The Yale-Brown Obsessive Compulsive Scale. I. Development, use, and reliability. Archives of General Psychiatry. 1989;46(11):1006-11.

14. Abramovitch A, Abramowitz JS, McKay D. The OCI-12: A syndromally valid modification of the obsessive-compulsive inventory-revised. Psychiatry Research. 2021;298:113808.

15. Kroenke K, Spitzer RL, Williams JB. The PHQ-9: validity of a brief depression severity measure. Journal of General Internal Medicine. 2001;16(9):606-13.

16. Beard C, Hsu KJ, Rifkin LS, Busch AB, Björgvinsson T. Validation of the PHQ-9 in a psychiatric sample. J Affect Disord. 2016;193:267-73.

17. Mundt JC, Marks IM, Shear MK, Greist JH. The Work and Social Adjustment Scale: a simple measure of impairment in functioning. Br J Psychiatry. 2002;180:461-4.

18. Rabin R, de Charro F. EQ-5D: a measure of health status from the EuroQol Group. Ann Med. 2001;33(5):337-43.

19. Oppe M, Devlin NJ, Szende A. EQ-5D value sets: inventory, comparative review and user guide: Springer; 2007.

**Supplementary Tables and Figures**

**Supplementary Table 1.** Content of the educational group sessions.

| **Session number and main session topic** | **Summarized content** | **Homework/Worksheet** |
| --- | --- | --- |
| 1. OCD education | OCD education (e.g., features, co-occurring conditions, risk factors). How OCD can have a deleterious impact on lifestyle (e.g., obsessions may lead to being housebound and not practicing regular exercise). | - Register OCD symptoms and how they affect lifestyle factors.  - Register daily physical activity and food intake. |
| 1. Overall lifestyle and health | What are unhealthy lifestyles. Advice on how to replace them with healthier choices. | - Decide on and try at least one healthier choice (e.g., take the stairs instead of the elevator, walk/cycle instead of driving, change to a low-fat dairy product or a non-sweetened product).  - Register daily physical activity and food intake. |
| 1. Physical activity and sedentary behavior | Definition of sedentary behavior and physical activity. Health benefits of exercise. Advice to avoid sedentary behavior and increase physical activity. | - Register daily physical activity and food intake. |
| 1. Food habits and alcohol use | Advice on healthy dietary habits based on the Nordic Nutrition Recommendations (2012): a dietary pattern rich in vegetables, fruits, fiber, and reduced saturated fats, salt, and red meat.  Risks of alcohol consumption and advice to quit drinking. | - Decide on and try at least one healthier food choice (e.g., change to a low-fat dairy product or a non-sweetened product, increase vegetable or fruit intake).  - Register daily physical activity, food intake, and alcohol consumption. |
| 1. Smoking, stress, and sleep | Advice on how to stop smoking (if applicable to any group members).  Anti-stress methods (e.g., breathing exercises, relaxation) and effects on cardiovascular risk will be introduced.  Sleep disorders and negative impact on health will be addressed. Psychoeducation on sleep hygiene. | - For smokers: Follow the recommendations from the Swedish Quit Smoking Line (<https://slutarokalinjen.se>).  - Practice of breathing and relaxation.  - Changes to improve sleep habits, if necessary.  - Register daily physical activity, food intake, and alcohol consumption. |
| 1. Behavioral change | Practical advice regarding change, motivation, and failure.  Goals setting and relapse prevention. | - Create a plan to maintain a healthier lifestyle. |

**Supplementary Table 2.** Number of cardiometabolic risk factors at baseline for each participant (n=25).

| **#** | **Low physical activity** | **Unhealthy diet** | **Risk consump-tion of alcohol** | **Tobacco use** | **Cardio-vascular disorder** | **Abdominal obesity** | **Over-weight** | **Hyper-tension** | **Dyslipi-demia** | **Impaired glucose tolerance** | **Type 2 diabetes mellitus** | **Sum of risk factors** |
| --- | --- | --- | --- | --- | --- | --- | --- | --- | --- | --- | --- | --- |
|  |  |  |  |  |  |  |  |  |  |  |  |  |
| 001 |  |  | X | X |  |  |  |  | X |  |  | 3 |
| 002 |  | X | X |  |  | X | X | X | X |  |  | 6 |
| 003 | X |  |  | X |  | X | X | X |  |  |  | 5 |
| 004 | X |  |  |  |  | X | X |  |  |  |  | 3 |
| 005 | X |  |  | X |  | X | X | X | X |  | X | 7 |
| 006 | X | X |  |  |  | X | X |  |  |  |  | 4 |
| 007 |  |  | X |  |  | X | X |  | X |  |  | 4 |
| 008 | X | X |  | X |  | X | X |  | X |  |  | 6 |
| 009 | X | X |  |  |  | X | X |  | X |  |  | 5 |
| 010 | X | X |  |  |  | X | X |  |  |  |  | 4 |
| 011 |  | X | X |  |  | X | X |  |  |  |  | 4 |
| 012 |  | X |  |  |  | X | X |  |  |  |  | 3 |
| 013 | X | X |  |  | X | X | X | X |  |  | X | 7 |
| 014 | X |  |  | X |  | X | X |  |  |  |  | 4 |
| 015 |  | X |  |  |  | X | X |  | X |  |  | 4 |
| 016 |  | X |  |  |  | X | X |  | X |  |  | 4 |
| 017 | X |  |  |  |  | X | X |  | X |  |  | 4 |
| 018 |  | X |  |  |  | X | X |  |  |  |  | 3 |
| 019 |  | X | X |  |  | X | X |  | X |  |  | 5 |
| 020 | X | X | X |  |  | X | X |  |  |  | X | 6 |
| 021 |  | X |  |  |  | X | X |  | X |  |  | 4 |
| 022 |  |  | X |  |  | X | X |  |  |  |  | 3 |
| 023 | X |  |  | X |  | X | X | X | X |  |  | 6 |
| 024 |  |  | X |  |  | X | X |  | X |  |  | 4 |
| 025 | X | X |  |  |  | X | X |  |  |  |  | 4 |
|  |  |  |  |  |  |  |  |  |  |  |  |  |
| n | 13 | 15 | 8 | 6 | 1 | 24 | 24 | 5 | 13 | 0 | 3 |  |
| % | 52 | 60 | 32 | 24 | 4 | 96 | 96 | 20 | 52 | 0 | 12 |  |
| Mean (*SD*) |  |  |  |  |  |  |  |  |  |  |  | 4.48 (1.23) |

**Supplementary Table 3.** Feasibility measures: Intervention credibility and intervention satisfaction.

| **Intervention credibility, n=25** | **M (SD)** |
| --- | --- |
| How well suited is the intervention for individuals with OCD to change their lifestyle habits?  (0 = not at all, 4 = very well) | 3.04 (0.89) |
| How much improvement do you expect from participating in the intervention?  (0 = none, 4 = much) | 2.80 (0.87) |
| How motivated are you to participate in the intervention?  (0 = not at all, 4 = very motivated) | 3.36 (0.95) |
| **Intervention satisfaction (Client Satisfaction Questionnaire-8), n=20** | **M (SD)** |
| How would you rate the quality of the intervention you have received?  (1= poor, 4 = excellent) | 3.40 (0.68) |
| Did you get the help you wanted?  (1 = no, definitely not, 4 = yes, definitely) | 3.20 (0.62) |
| To what extent has the intervention met your needs?  (1= none of my needs have been met, 4 = almost all of my needs have been met) | 3.10 (0.79) |
| If a friend were in need of similar help, would you recommend the intervention to him or her?  (1 = no, definitely not, 4 = yes, definitely) | 3.70 (0.57) |
| How satisfied are you with the amount of help you received?  (1 = quite dissatisfied, 4 = very satisfied) | 3.05 (0.69) |
| Has the intervention you received helped you to deal more effectively with your problems?  (1 = no, they seemed to make things worse 4 =yes, they helped a great deal) | 3.15 (0.59) |
| Overall, how satisfied are you with the intervention you received?  (1 = quite dissatisfied, 4 = very satisfied) | 3.25 (0.72) |
| If you were to seek help again, would you seek a similar intervention?  (1 = no, definitely not, 4 = yes, definitely) | 3.50 (0.76) |
| *Total score* (maximum 32 points) | 26.35 (4.31) |

**Supplementary Table 4.** Self-reported adverse events on a scale from 0 = not at all to 4 = persistently.

| **Adverse events, mid-intervention (n = 10)** | **M (SD)** |
| --- | --- |
| Loss of appetite | 0.40 (0.52) |
| Irritable | 0.40 (0.70) |
| Stomach pain | 0.50 (0.71) |
| Headache | 0.89 (0.78) |
| Sleep difficulties | 0.56 (1.33) |
| Muscle pain | 1.40 (1.07) |
| Stiffness | 0.80 (0.92) |
| Increased anxiety | 0.80 (1.23) |
| Increased obsessions | 0.80 (1.23) |
| Increased stress | 0.80 (1.23) |
| Dizziness | 0.50 (0.97) |
| Fatigue | 1.10 (0.99) |
| **Adverse events, post-intervention (n = 20)** | **M (SD)** |
| Loss of appetite | 0.35 (0.67) |
| Irritable | 0.55 (0.83) |
| Stomach pain | 0.30 (0.57) |
| Headache | 0.70 (1.17) |
| Sleep difficulties | 0.35 (0.59) |
| Muscle pain | 1 .00 (1.26) |
| Stiffness | 0.90 (1.21) |
| Increased anxiety | 0.80 (1.11) |
| Increased obsessions | 0.65 (0.99) |
| Increased stress | 1.00 (0.88) |
| Dizziness | 0.25 (0.72) |
| Fatigue | 1.30 (2.49) |

**Supplementary Table 5.** Raw means and standard deviations for all measures at baseline, post-intervention, and 3-month follow-up.

| **Measures** | **Baseline**  **(n=25)** | **Post-intervention**  **(n=21)** | **3-month follow-up**  **(n=21)** |
| --- | --- | --- | --- |
|  | **M (*SD*)** | **M (*SD*)** | **M (*SD*)** |
| **Lifestyle habits** |  |  |  |
| Physical activity (IPAQ MET) | 912.80 (1100.69) | 1481.238 (2056.571) | 1293.95 (1800.86)^d^ |
| Sedentary time (hours) | 8.48 (4.29)^a^ | 7.48 (5.69) | 9.44 (6.13)^e^ |
| Physical activity (steps per day) | 4762.03 (3067.85)^b^ | 4837.98 (3697.81)^e^ | 5059.37 (3287.79)^c^ |
| Dietary habits (Dietary index score) | 4.08 (2.00) | 5.24 (2.28) | 5.19 (2.23) |
| Alcohol use (AUDIT-C) | 2.84 (2.72) | 2.14 (2.22) | 1.86 (1.77) |
| Tobacco use (n, %) | 6 (24.00) | 4 (19.05) | 4 (19.05) |
| Stress (PSS) | 24.92 (7.53) | 21.86 (7.14) | 22.24 (7.69) |
| Sleep (ISI) | 13.52 (7.14) | 11.70 (6.47)^c^ | 11.76 (7.69) |
| **Anthropometric measures** |  |  |  |
| Weight (kg) | 95.64 (18.77) | 97.05 (18.12) | 98.41 (18.53) |
| BMI (kg/m^2^) | 32.84 (5.76) | 33.27 (5.28) | 33.73 (5.31) |
| Waist circumference (cm) | 108.46 (14.94) | 109.26 (13.55) | 109.69 (13.04) |
| Sagittal abdominal diameter (cm) | 27.32 (4.29) | 27.12 (4.33) | 28.12 (4.41) |
| Total body fat (%) | 37.38 (8.03) | 38.30 (6.53) | 38.24 (6.39) |
| Fat around the waist (%) | 35.16 (6.19) | 36.58 (4.96) | 37.57 (5.98) |
| Systolic blood pressure (mmHG) | 120.48 (13.02) | 120.50 (11.94) | 118.69 (9.65) |
| Diastolic blood pressure (mmHG) | 79.84 (6.32) | 78.14 (6.85) | 79.43 (5.87) |
| Resting heart rate | 76.14 (10.58) | 72.60 (9.63) | 76.24 (15.85) |
| Framingham risk score | 3.77 (3.81) | 3.84 (3.65) | 3.84 (3.17)^c^ |
| **Blood samples** |  |  |  |
| Total cholesterol (mmol/L) | 4.83 (0.96) | 4.80 (1.14) | 5.10 (1.19)^c^ |
| LDL-cholesterol (mmol/L) | 2.96 (0.83) | 2.95 (0.96) | 3.06 (0.95)^d^ |
| HDL-cholesterol (mmol/L) | 1.32 (0.42) | 1.32 (0.36) | 1.31 (0.35)^c^ |
| Triglycerides (mmol/L) | 1.19 (0.72) | 1.20 (0.75) | 1.52 (0.97)^c^ |
| Glucose (mmol/L) | 5.32 (0.41) | 5.47 (0.41) | 5.6 (0.54)^c^ |
| HbA1c (mmol/L) | 34.48 (3.44) | 35.57 (3.85) | 35.15 (4.15)^c^ |
| C-reactive protein | 4.67 (6.78) | 5.20 (6.54) | 3.72 (3.26)^c^ |
| 2h-oral glucose tolerance test | 5.14 (1.73)^a^ |  | 5.65 (1.09)^f^ |
| **Mental health and quality of life** |  |  |  |
| Y-BOCS | 23.48 (5.99) | 19.24 (5.76) | 19.86 (7.09) |
| OCI-12 | 24.84 (9.62) | 22.57 (10.07) | 23.95 (10.84) |
| PHQ-9 | 12.92 (6.49) | 10.19 (5.86) | 11.00 (6.84) |
| WSAS | 20.48 (11.17) | 19.81 (10.61) | 17.62 (11.17) |
| EQ-5D-3L index score | 0.43 (0.31) | 0.61 (0.23) | 0.53 (0.29) |
| EQ-5D-3L visual analogue scale | 46.28 (20.44) | 53.29 (17.85) | 50.62 (18.37) |

*Abbreviations:* AUDIT-C=Alcohol Use Disorders Identification Test; BMI=Body Mass Index; EQ-5D-3L=The EuroQol five dimensional three level questionnaire; HbA1c= glycated hemoglobin; HDL= high-density lipoprotein; ISI= Insomnia Severity Scale; IPAQ= International Physical Activity Questionnaire; LDL= low-density lipoprotein; MET= Metabolic equivalent of task; OCI- 12=Obsessive Compulsive Inventory; PHQ-9=Patient Health Questionnaire; PSS-10=Perceived Stress Scale; Y-BOCS= Yale-Brown Obsessive-Compulsive Scale; WSAS= Work and Social Adjustment Scale.

a Based on n=23; b Based on n=22; c Based on n=20; d Based on n=19; e Based on n=18; f Based on n=13

**Supplementary Figure 1.** Participants’ attendance to a) educational group sessions, and b) exercise group sessions (n=25).
